# The impact of antiretroviral therapy on population-level virulence evolution of HIV-1

**DOI:** 10.1098/rsif.2015.0888

**Published:** 2015-12-06

**Authors:** Hannah E. Roberts, Philip J. R. Goulder, Angela R. McLean

**Affiliations:** 1 Nuffield Department of Clinical Medicine, The Peter Medawar Building for Pathogen Research, University of Oxford, Oxford OX1 3SY, UK; 2 Department of Paediatrics, University of Oxford, Oxford OX1 3SY, UK; 3 HIV Pathogenesis Programme, The Doris Duke Medical Research Institute, University of KwaZulu-Natal, Durban, South Africa; 4 The Institute for Emerging Infections, The Oxford Martin School, Oxford OX1 3BD, UK; 5 Department of Zoology, University of Oxford, Oxford OX1 3PS, UK

**Keywords:** HIV-1, virulence evolution, antiretroviral therapy, set point viral load, between-host modelling

## Abstract

In HIV-infected patients, an individual's set point viral load (SPVL) strongly predicts disease progression. Some think that SPVL is evolving, indicating that the virulence of the virus may be changing, but the data are not consistent. In addition, the widespread use of antiretroviral therapy (ART) has the potential to drive virulence evolution. We develop a simple deterministic model designed to answer the following questions: what are the expected patterns of virulence change in the initial decades of an epidemic? Could administration of ART drive changes in virulence evolution and, what is the potential size and direction of this effect? We find that even without ART we would not expect monotonic changes in average virulence. Transient decreases in virulence following the peak of an epidemic are *not* necessarily indicative of eventual evolution to avirulence. In the short term, we would expect widespread ART to cause limited downward pressure on virulence. In the long term, the direction of the effect is determined by a threshold condition, which we define. We conclude that, given the surpassing benefits of ART to the individual and in reducing onward transmission, virulence evolution considerations need have little bearing on how we treat.

## 1. Introduction

During a typical HIV-1 infection, viral load rises sharply to a peak in the first few weeks, then decreases to the set point viral load (SPVL) and is relatively stable thereafter [1]. The end of this long period of chronic asymptomatic infection is marked by an increase in viral load and progression to AIDS [2]. The term ‘virulence’ relates to a microorganism's capacity to cause disease and so for HIV, virulence is defined as the rate of progression to AIDS [3,4].

Time between seroconversion and progression to AIDS varies greatly between HIV-1 infected individuals. Some experience rapid CD4^+^ T-cell decline and develop AIDS within a few months or years, whereas others remain AIDS-free for over 35 years [5,6]. It is likely that the course of disease within an individual is determined by an interplay between host factors (including host genetics) and viral genetic factors [5,7]. There is ongoing debate about how much of the variation in time to progression can be attributed to each set of factors but recently the nature of the role played by virus genetics has received particular attention [8,9].

HIV-1 is a relatively recent infection of humans, having crossed over from non-human primates in the last century [10], and it is likely to still be adapting to its new host. As is often the case with host–parasite interactions, there are constraints upon the virulence evolution of HIV. The level of SPVL is highly predictive of time to AIDS [11] but high SPVL is also linked to an increased risk of transmission during chronic infection [12]. The proportion of transmissions that occur during acute versus chronic infection probably varies between settings [13,14] but, given revised estimates of infectivity during the acute phase, it seems reasonable to expect that the majority of transmissions occur during chronic infection (see [14] for a full discussion). Therefore, the evolutionarily optimal value of SPVL balances the duration of infectiousness and transmission rate, a concept termed the ‘trade-off hypothesis' [15]. The trade-off hypothesis has been applied previously to the specific problem of HIV virulence evolution, with SPVL used as a marker for virulence [16].

While there has been much work looking at whether the virulence of HIV-1 might be changing [3], there has been less on the possible impact of antiretroviral therapy (ART) on virulence evolution. Yet given the widespread use of ART and the striking reduction in infectiousness of ART-treated patients [17,18], there is clearly the potential for ART to have evolutionary consequences beyond those of drug resistance. Coupled with the fact that patients are not treated at random but often according to their CD4 count, ART could be an added selection pressure on the evolution of HIV virulence. Indeed, it has been speculated that widespread ART could drive either increases [7,19] or decreases [4] in virulence. The benefits of ART to the individual and to epidemic control are so spectacular that they are going to be paramount in importance to any effects of ART on virulence evolution. However, the epidemic is still far from over and increased coverage of ART does not yet imply the end of HIV transmission [20]. Hence it is still important to understand what might be the implications of widespread ART for future patterns of virulence change.

In this paper, we first examine the observed patterns of changing HIV virulence, and then introduce two models to answer the following questions; in the absence of ART, how would we expect the virulence of a novel infection to change? Would we expect widespread ART to drive changes in HIV virulence evolution, and if so, what is the expected magnitude and direction of any ART effect? Our analysis is based on an ODE model and a PDE model where time since infection is introduced as a second independent variable and which is equivalent to the ODE model at epidemic equilibrium. For clarity in the main text, we start with the simplest form of the model and build it up in stages. The PDE model is employed specifically to help our understanding of the equilibrium effects of ART on population average SPVL.

## 2. Modelling and results

### 2.1. What are the observed patterns of changing HIV virulence?

**Figure 1.**
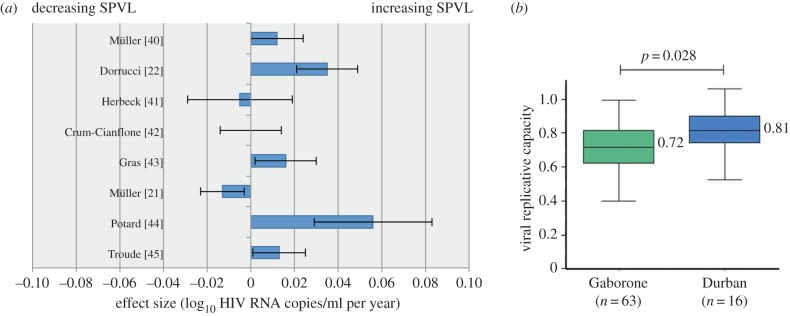
A collection of empirical data, reproduced from a meta-analysis by Herbeck *et al.* [3] (*a*) and from Payne *et al.* [4] (*b*). (*a*) Results from eight studies measuring population-level trends in SPVL [21–28], showing significant trends in both directions. The effect sizes, i.e. the estimated per-year change in SPVL, and 95% CIs (error bars) are shown. Criteria for inclusion of these studies in the meta-analysis were that they controlled for ART use and reported data with which regression slopes could be estimated. Herbeck *et al.* calculated a summary estimate using a weighted least-squares approach as 0.013 (95% CI [−0.001,0.027]), full details in [3]. (*b*) The VRC of viruses isolated from mothers in Gaberone compared with Durban is significantly lower in Gaberone where the epidemic is around 10 years older and ART was introduced 5 years earlier. VRC was measured in identical conditions and normalized to NL4-3 comparator virus for these CD4-matched groups (CD4 300–500 mm^–3^).

Individual studies based on measurement of either baseline CD4 count or SPVL have reported statistically significant trends in virulence in both directions [21,22]. The overall picture is confounded by the differing measures of virulence used, substantial changes in standards of assays, possible sampling biases and the distinct character of separate sub-epidemics. Even just restricting analyses to SPVL, the pattern is unclear (figure 1*a*). However, there is an emerging consensus that in the past few decades there has been an overall increase in SPVL across HIV-1 infected populations with a majority European ancestry [3,29]. In contrast, in a recent study of HIV-1-infected mothers in Botswana and South Africa, data describing viral replicative capacity (VRC), an *in vitro* measure of viral fitness, pointed to a decrease in virulence over time. In Gaberone, Botswana, where the epidemic is 10 years older, median VRC of the cohort was significantly lower than in Durban, South Africa (figure 1*b*). We will refer back to these data later when we come to set our modelling results in the context of observed trends.

### 2.2. In the absence of treatment, how would we expect the virulence of a novel infection to change?

**Figure 2.**
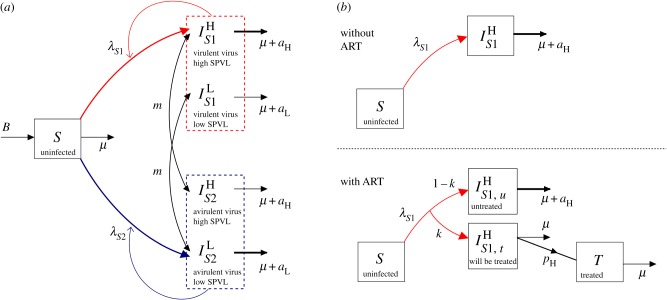
A model diagram, (*a*) for the basic model in the absence of ART (equations (2.1)) and (*b*) showing the incorporation of ART into the model (equations (2.2)). (*a*) Uninfected individuals, *S*, enter the population at rate *B* and die at rate *μ*. They may be infected with either strain, *S*1 (red) or *S*2 (blue). All infections initiated by strain *S*1 have high SPVL (H) and all infections initiated by *S*2 have low SPVL (L). Within host strain switching occurs at a low rate, *m*, and SPVL within that host is preserved when this happens. The force of infection for each strain is given by 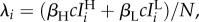 where 
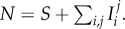
 Infected individuals have an accelerated death rate due to AIDS of *μ* + *a_j_*, where *a*_H_ > *a*_L_. (*b*) Use of ART at 100*k*% coverage is reflected by a proportion *k* of individuals entering the ‘will be treated’ class upon infection and progressing on to treatment at rate *p_j_*, where *p*_H_ > *p*_L_. Treated individuals, *T*, are assumed to be non-infectious and die at rate *μ*. Model parameters are fully described in table 1.

There is a received wisdom that successful parasites are relatively harmless to their hosts, and therefore that new infectious diseases of humans will evolve to avirulence. This general assumption has been challenged by the ‘trade-off hypothesis’—virulence is often linked to the chance of onward transmission, as is the case with HIV, and hence the ‘optimal’ level of virulence must balance transmissibility and duration of infectiousness [15,30]. We have encapsulated this virulence trade-off in a simple two-strain model. For simplicity, we assume that the SPVL of a new infection is entirely determined by the genotype of the infecting strain (analyses with this assumption relaxed are shown in electronic supplementary material, §2). Accordingly, infection with strain 1 results in high SPVL infections which have both a high transmission coefficient (*β*_H_*c*) and high AIDS-related death rate (*a*_H_). Infection with strain 2 results in low SPVL infections with low transmission coefficient (*β*_L_*c*) and AIDS-related death rate (*a*_L_). The two strains are in competition and low numbers of infections of both are introduced into an initially susceptible population. Strain switching within host occurs at a low rate, *m*, to avoid one strain necessarily becoming extinct, and SPVL is preserved within that host when this happens. Hence, an individual's disease course and infectiousness is determined entirely by the infecting strain, but strain switching can lead to them passing on the opposite strain. The model equations, in the absence of ART, are2.1
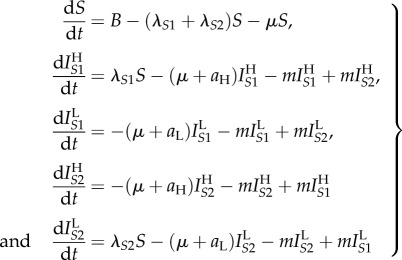
where *S* represents susceptible (uninfected) individuals and the 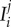 represent the different infected classes, where *i* denotes strain type and *j* denotes SPVL (H = high and L = low). All new infections are either in class 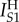 or 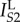 but, partway through an infection, strain switching can lead to these switching to become 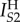 and 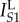 infections, respectively. The parameters are fully described in table 1. Briefly, *B* is the rate at which new susceptibles are added to the population, *μ* is the death rate of uninfected individuals and 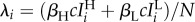 is the force of infection of each strain (where 
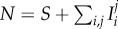
). A model diagram is shown in figure 2*a*. As *m* is small, the *R*_0_s for both strains are approximated by
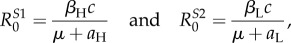
(this calculation is exact when *m* = 0).

**Table 1.** Parameter values and references.

symbol	description	value used	interpretation
*μ*	death rate of uninfected individuals	1/40	the life expectancy of an HIV-uninfected individual in the population is on average 40 years, after reaching adulthood. This estimate is based on the WHO African Region [17]
*B*	population birth rate	*µS*(0)	the population size is constant in the absence of infection
*a*_H_, *a*_L_	AIDS-related death rates of high and low (respectively) SPVL infections	1/5, 1/11	in the model, an infected individual with high SPVL dies on average 5 years after infection, an infected individual with low SPVL dies on average 11 years after infection. In an HIV-infected Ugandan population, 81% survived 5 years post-seroconversion and 48% survived 10 years post-seroconversion [31]
*p*_H_, *p*_L_	rates at which high and low (respectively) SPVL infections progress to the CD4 count threshold for commencing ART	in figure 3, these are set at 1/4 and 1/10. In figures 5 and 6, *p*_H_ and *p*_L_ vary	in figure 3, patients are treated on average a year before they would otherwise have died of AIDS. In figures 5 and 6, the ‘low CD4’ period is at least a year. The lower bound estimate of 1 year for the ‘low CD4’ period is based on rates of CD4 count decline [32] and CD4 count treatment thresholds of 200 cells per mm^3^ or greater [33]
*β*_H_*c*, *β*_L_*c*	transmission coefficients of high and low (respectively) SPVL infections. Composed of *c* (contact rate) and *β_j_* (probability of transmission upon contact of infectious and non-infectious individuals)	≈0.52, ≈0.26	values are chosen to yield 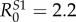 and 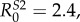 or vice versa, in an untreated population. This puts the peak of the epidemic at 40 years in, consistent with estimates from Botswana and South Africa where epidemic emergence occurred in the 1950s–1960s [34] and the epidemics peaked around 2000–2005 [35]. A wider range of *R*_0_ values is considered in electronic supplementary material, figures S11–S13
*k*	ART coverage	in figure 3, *k* = 0.3. In figures 5 and 6, *k* varies.	from 40 years into the epidemic 100*k*% of new infections will eventually receive treatment [17]. The introduction of ART coincides with the peak of the epidemic, reflecting the situation in South Africa [35,36]. Changing the timing of ART introduction relative to the peak has a negligible effect on virulence evolution (electronic supplementary material, figures S14 and S15)
*m*	strain switching rate	0.001	strain switching will occur in approximately 1 in every 1000 infections each year. We choose *m* > 0 simply so that both strains can be present at equilibrium.

**Figure 3.**
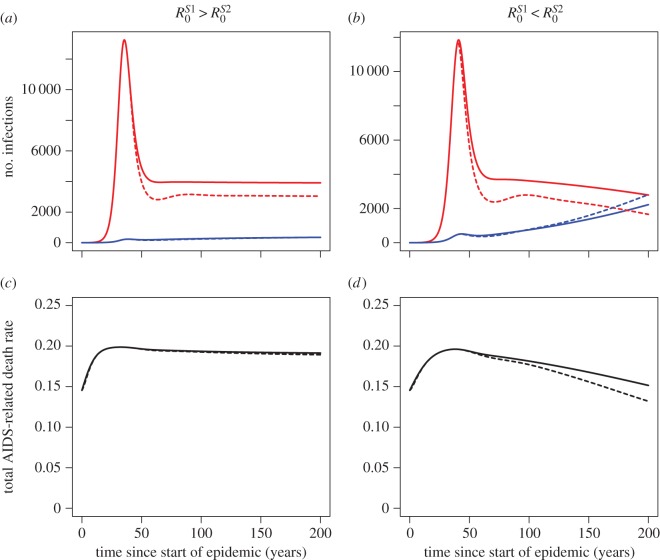
Behaviour of the two strain model with and without ART. (*a*,*c*) The transmission coefficients, *β*_H_*c* and *β*_L_*c*, are chosen so that in the absence of ART *S*1 will dominate at equilibrium (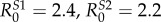). (*b*,*d*) *β*_H_*c* and *β*_L_*c* chosen so that in the absence of ART *S*2 will dominate at equilibrium (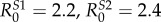). The initial conditions are 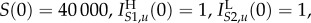 all other populations equal to zero. The remaining parameter values are given in table 1. Solid lines show results of the model simulation with no ART, dotted lines show the results when ART is introduced at 30% coverage 40 years into the epidemic. (*a*,*b*) The numbers of strain *S*1 (red) and strain *S*2 (blue) infections during the course of the epidemic. (*c*,*d*) Average virulence, measured in terms of the total number of AIDS-related deaths per untreated infected person per year and calculated as 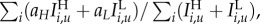 through time. Early in the epidemic the spread of infection is dominated by the more virulent strain because it has a higher force of infection. However, the slow growth of the less virulent strain and peak of virulent infections (*a*,*b*) leads to a second phase during which average virulence falls (*c*,*d*). Simulations of the model with a range of initial conditions and spanning the first 1000 years of the epidemic are shown in electronic supplementary material, figure S16.

Model simulations of the epidemic with initial conditions 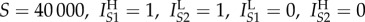 are shown in figure 3 (solid lines). Note that the underlying population size makes no difference to the dynamics of our model because we have used the standard assumption that transmission of a sexually transmitted disease is proportional to *βSI*/*N* [15]. We simulate two scenarios: 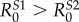 (figure 3*a,c*) and 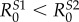 (figure 3*b*,*d*). Throughout the results, the number of AIDS-related deaths per untreated infected person per year is used as a proxy for virulence (figure 3*c*,*d*).

In the first few decades of the epidemic, the virulent strain (*S*1) spreads faster through the population due to its high transmissibility and hence dominates. Following this, the slow growth of the low virulence strain (*S*2) and peaking of *S*1 infections leads to a second phase in which average virulence falls. The long-term equilibrium behaviour of the model constitutes a third phase in which the strain balance is determined by the *R*_0_*s* (the whole of the third phase can be seen in electronic supplementary material, figure S16). Because equilibrium is not reached for hundreds of years, the decline of virulence during the second phase is seen clearly whatever the balance of the *R*_0_*s* (figure 3*c*,*d*). Even when the virulence/transmissibility trade-off means that *S*1 will predominate in the long run, the proportion of infections caused by this higher virulence strain still decreases for a while after the peak. The characteristic first, second and third phase behaviour described above is robust to changes in the *R*_0_ values and initial conditions of the model (electronic supplementary material, figures S11–S13 and S16). The values used in figure 3 were chosen because they produce results that are representative of the middle of the range.

### 2.3. Would we expect widespread antiretroviral therapy to drive changes in HIV virulence evolution?

We incorporate ART into the model by supposing that a proportion, *k*, of individuals will eventually be treated, with the rest remaining untreated until death. Therefore, we split each infected class, 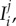 into 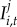 (treated) and 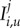 (untreated), with 
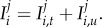
 The extended set of model equations is2.2
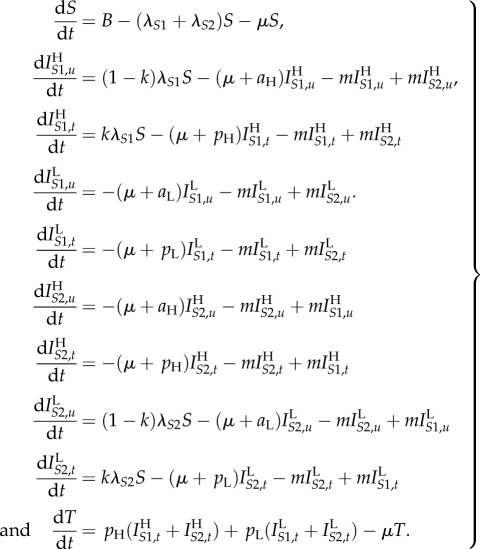


Once treated, patients are assumed to become non-infectious. We model treating based on CD4 count [33], by assuming that individuals with high SPVL will be treated sooner than those with low SPVL, at rates *p*_H_ and *p*_L_, respectively (*p*_H_ > *p*_L_). See figure 2*b* for a model diagram.

In figure 3, we see that introducing ART 40 years into the epidemic (to roughly coincide with the peak, as in data from South Africa; table 1) slightly decreases virulence in the decades after it is introduced. We ask, is this indicative of a general pattern?

Intuitively, we might expect treatment based on patient CD4 count to favour less virulent viruses since it is only the most sick patients that get treated. Expressing this intuitive argument formally allows us to examine exactly when this is true: *if* a randomly chosen infected person with ‘low’ CD4 count (i.e. a person eligible for treatment) is more likely to be infected with virulent virus than a person chosen at random from the entire infected pool, then treating ‘low’ CD4 count patients will favour the less virulent virus. This ‘if’ clause holds when the following inequality is satisfied:2.3
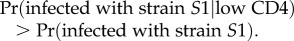


In order to calculate the sizes of the infected, ‘low CD4’ populations at equilibrium for both strains, we now introduce an infection-age-dependent model (which we will refer to as the ‘PDE model’) and relate it back to the original ODE model.

### 2.4. Defining the infection-age-dependent model

**Figure 4.**
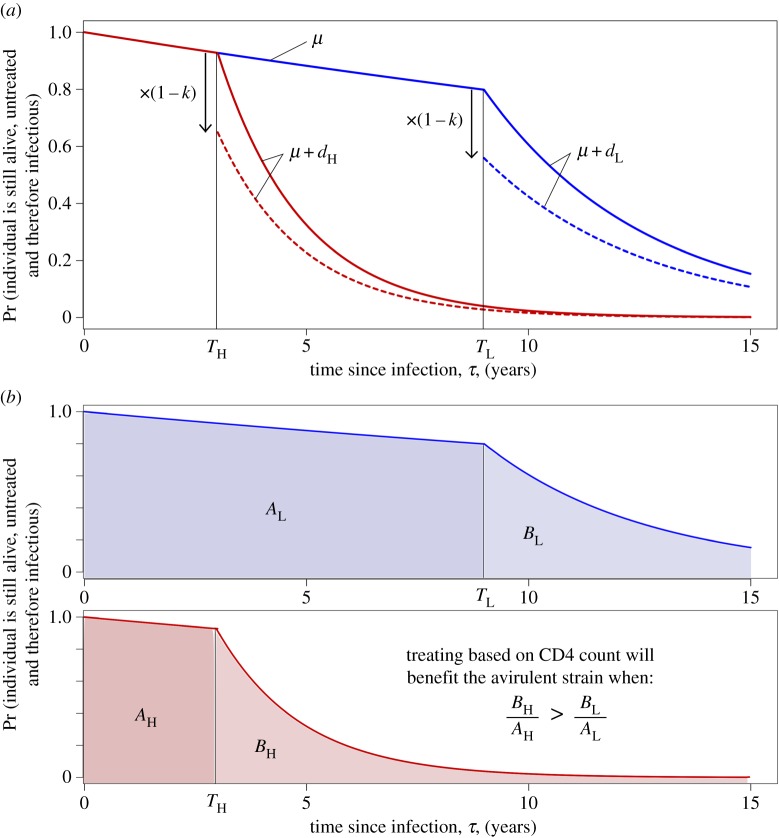
Diagrams representing the PDE version of the model and the relative importance of low CD4 transmissions. As in figure 3, the high virulence strain (*S*1) is shown in red and the low virulence strain (*S*2) in blue. We here assume *S*1 infections always have high SPVL and *S*2 infections always have low SPVL (i.e. *m* = 0, as in the PDE model calculations in the main text). The graphs show the probability that an individual will still be alive and untreated and hence infectious *τ* years into their infection, plotted against *τ*. (*a*) In the PDE model, AIDS-related death of an individual is dependent on time since infection and only occurs after the low CD4 threshold has been crossed. A proportion, *k*, of treated patients is removed from the infectious class as they reach this threshold, giving rise to the discontinuity. Solid line, no ART; dotted line, ART at 30% coverage. (*b*) Similar to (*a*) but split by strain. Shaded areas are proportional to the contribution of that period of the infection to onward transmissions of that strain in a model without treatment. When patients are treated they are no longer infectious (after *T_j_*) and the same proportion are treated for each strain, therefore the effect of ART on the strain balance at equilibrium is determined by the relative importance of low CD4 transmissions, *B*_H_/*A*_H_ compared with *B*_L_/*A*_L_.

Let *τ* denote time since infection, and define 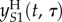 to be the number of high SPVL, virulent strain (*S*1) infections of infection-age *τ* at time *t*, so that
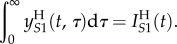
We assume that patient CD4 count drops below the ‘low’ threshold *T*_H_ years after infection with *S*1 or *T*_L_ years after infection with *S*2 (*T*_L_ > *T*_H_)). In this model, the death rate is dependent on time since infection; the parameter *d_j_* is the additional AIDS-related death rate of patients who have crossed the low CD4 count threshold. A proportion, *k*, of patients are treated at the point (*τ* = *T_j_*) when they reach the low CD4 count threshold, resulting in a discontinuity in 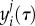 at *T_j_* (figure 4*a*). The model equations are as follows:
2.4
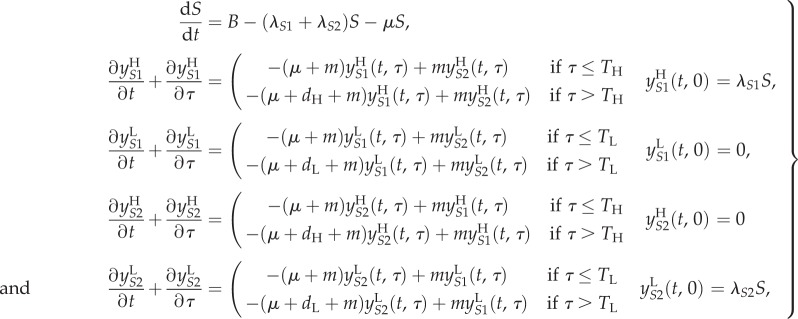


where *λ_S_*_1_ and *λ_S_*_2_ are defined as before. At equilibrium, when 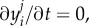 the above reduce to a set of ODEs in *τ* which we may solve to obtain 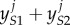 and 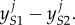 Combining these simultaneous equations appropriately yields
2.5
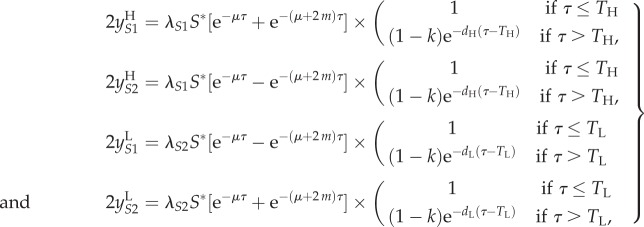


where S* is the equilibrium value of *S*, and so *λ_S_*_1_S* and *λ_S_*_2_S* are constants (independent of *τ*).

In applying the PDE model later on, it will be useful to be able to relate the parameters *T_j_* and *dj* to the parameters *a_j_* and *p_j_* in the ODE model. We may do this by equating the two models at equilibrium, i.e. ensuring that at equilibrium the sizes of both the high SPVL and low SPVL infected classes in the ODE model match those in the PDE model. Hence, we require that, for *j* = *H*, *L*:
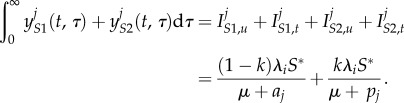


After substituting in the solutions to the PDE model at equilibrium in (2.5) and integrating, this reduces to
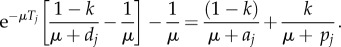


Substituting in *k* = 0 and *k* = 1 shows that in order to obtain a condition that is independent of *k* we must have 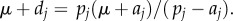 In this case, the above simplifies to 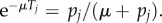


In conclusion, the parameters in the two models are related according to
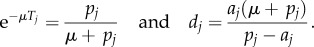


A simple example shows that the parameters seem to correspond as we would expect: take *µ* = 1/40, *T_j_* = 4 years (i.e. time to reaching ‘low CD4’ treatment threshold), *d_j_* = 1 per year (i.e. AIDS-related death rate), then the calculations give *p_j_* ≈ 9/40 and *a_j_* ≈ 9/50. So average time to progression is just over 4 years and average time to death in an untreated patient is just over 5 years.

### 2.5. When does antiretroviral therapy favour less virulent viruses?

We now apply this infection-age-dependent model to calculating when the inequality in (2.3) holds. In terms of the populations in the infection-age-dependent model, (2.3) is equivalent to
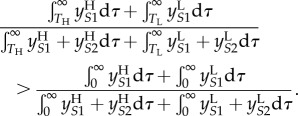


Assuming that the strain switching rate, *m*, is negligible, so that 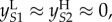 this becomes:2.6
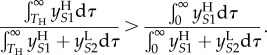


The integrals in this inequality are represented graphically in figure 4*b*. In the notation of the figure, inequality (2.6) is equivalent to2.7
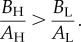


To describe this inequality in an intuitively appealing way, we note that it relates to ‘the relative importance of low CD4 transmissions' for each strain: when ART is introduced, a proportion, *k*, of transmissions caused by individuals who have passed beyond the low CD4 threshold are effectively removed for each strain. The strain that is most severely affected by ART will be the one for which these ‘low CD4’ transmissions made up the greatest proportion of the overall number of transmissions. Hence, we find that the intuition that treatment will favour the low virulence viruses is true in the long run (at equilibrium) in the case where the proportion of low CD4 transmissions (*B_i_*/*A_i_*) is greater for high SPVL infections. This is when (2.7) holds.

If expected survival time after progressing to ‘low CD4’ count is the same for high and low SPVL infections, or if it is longer for low SPVL infections but only in proportion to the additional time taken to reach the low CD4 threshold, then (2.7) holds. In these cases, the latter of which seems consistent with available data [32], treatment will favour the low virulence viruses. Indeed, for this not to hold it must be the case that individuals with low SPVL continue to survive with low CD4 count for a disproportionately long time (compared to individuals with low CD4 counts caused by high SPVL infections).

### 2.6. What is the expected magnitude and direction of this antiretroviral therapy effect?

The equilibrium calculations above are not the whole picture and the magnitude and direction of this ART effect may be influenced by considering the effect soon after the introduction of ART as opposed to at equilibrium. We may rewrite (2.6) in terms of the parameters of the PDE model by substituting in the solutions for the populations at equilibrium given in (2.5) (full workings in electronic supplementary material, §1.3):2.8
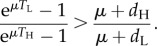


**Figure 5.**
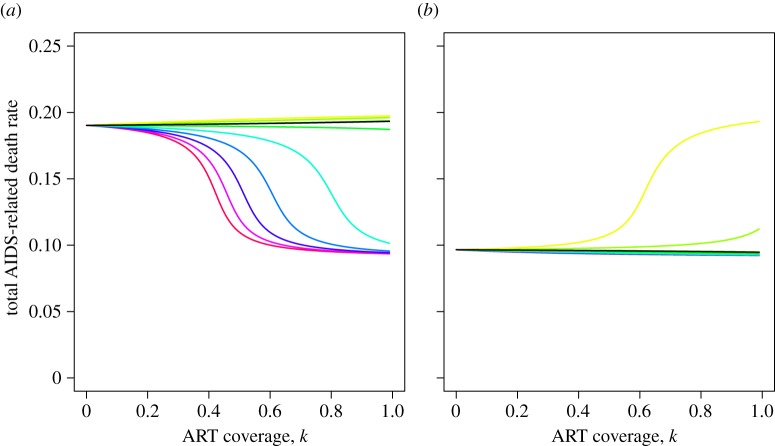
The relationship between ART coverage and virulence at equilibrium, for different parameter sets. (*a*) 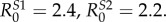 (*b*) 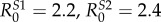 (*R*_0_s calculated in the absence of ART). Different coloured lines represent different proportions of *S*2 transmissions removed as a result of ART. So, with reference to figure 4*c*, *B*_H_/*A*_H_ is kept constant (parameters *T*_H_ = 3, *d*_H_ = 0.57) while *B*_L_/*A*_L_ is varied from low (red line; parameters *T*_L_ = 8, *d*_L_ = 0.57) to high (yellow line; parameters *T*_L_ = 5, *d*_L_ = 0.2). The ODE model parameters corresponding to these PDE model parameters were calculated as set out in the main text and then the equilibrium position of the ODE model was computed (electronic supplementary material, §1.4) to produce these plots. The black line corresponds to the parameter set in which ART has no effect at equilibrium (equality in (2.8)). Inequality (2.8) was obtained under the assumption that the switching rate, *m*, is negligible whereas these calculations of the equilibrium behaviour use *m* = 0.001, hence this black line deviates marginally from the horizontal. Total number of AIDS-related deaths per untreated infected person per year is calculated as in figure 3. The extreme values of this are *a*_H_ = 1/5 and *a*_L_ = 1/11 and these are approached when *S*1 and *S*2 (respectively) completely dominate at equilibrium. (*a*) In the absence of ART, *S*1 dominates at equilibrium. Hence, when *B*_L_/*A*_L_ < *B*_H_/*A*_H_ then ART is able to switch the ordering of the *R*_0_s so that *S*2 dominates at equilibrium. The more severe the inequality between *B*_L_/*A*_L_ and *B*_H_/*A*_H_, the lower the ART coverage needed to cause this switch. (*b*) In the absence of ART, *S*2 dominates at equilibrium, and so this situation is the reverse of (*a*).

**Figure 6.**
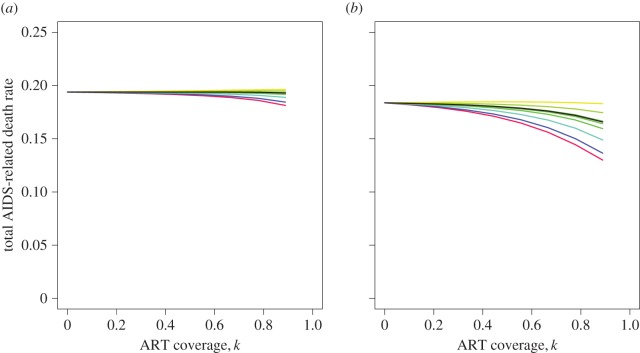
The effect of ART on virulence in the short term, 50 years after the introduction of ART. The results of model simulations, in which ART is introduced 40 years into the epidemic, are shown, with total number of AIDS-related deaths per untreated infected person per year once again used as a proxy for virulence. (*a*) 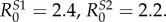 (*b*) 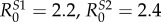 (*R*_0_s calculated in the absence of ART). Different coloured lines correspond to different sets of model parameters which determine the effect of ART on the balance of removed transmissions, as described in figure 5. Initial conditions and fixed parameters are as given in figure 3 and table 1. Note that points are not plotted for *k* = 1 because when all patients are treated there are no AIDS-related deaths and so overall virulence is not well defined at this point. The similarity of the results in (*a*) and (*b*) shows that changes in the balance of the *R*_0_ values have little effect on model behaviour 90 years into the epidemic. *S*1 dominates in both scenarios in the absence of ART and the effect of ART is, in almost all cases considered, to benefit the low virulence strain at this point. The ART effect is stronger for any given level of coverage when *B*_L_/*A*_L_ < *B*_H_/*A*_H_ (red-blue lines).

Then, using the relationship between the ODE and PDE model parameters, we are able to use the ODE model to confirm this analytical result at equilibrium (figure 5) and see how it compares to the short-term ART effect (figure 6).

Figure 5 shows the effect of ART on virulence at equilibrium for a range of different values of *T_j_* and *d_j_* which we believe to span the reasonable parameter space. As expected, the direction of the ART effect is determined by the relative importance of low CD4 transmissions. To understand the magnitude of the effect, note that the equilibrium virulence tends to rest at one of two extremes, determined by which strain dominates in the absence of ART. The values are extreme because the frequency of the subdominant strain is at mutation–selection balance which is very low when *m* is small, as here. Therefore, the magnitude of the ART effect is largely determined by whether there is potential for ART to cause the strain balance to switch to the other extreme. For example, when 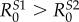 (figure 5*a*) then a large ART effect is only seen when ART exerts a downward pressure on virulence, i.e. when low CD4 transmissions are of greater importance to *S*1. These results are summarized in table 2.

**Table 2.** Summary results table. This table describes the magnitude and direction of the effect of ART on virulence depending on (i) whether it were to be measured soon after the introduction of ART (‘early’ = within 50 years) or centuries later (‘late’), (ii) the balance of the *R*_0_s of the two strains, and (iii) the relative importance of ‘low CD4’ transmissions to both strains.

	early		late	
	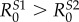	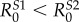	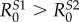	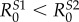
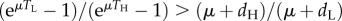	medium decrease in virulence	medium decrease in virulence	large decrease in virulence	small decrease in virulence
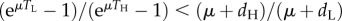	small decrease in virulence	small decrease in virulence	small increase in virulence	medium increase in virulence

Fifty years after the peak of the epidemic, the high virulence strain (which has the larger growth rate) always dominates in the absence of treatment (figure 6). The effect of ART seen at this time point is to decrease virulence for almost all sets of parameters considered. This transient decrease in virulence, which masks any selective pressure exerted by the ‘relative importance of low CD4 transmissions’, is a result of the more immediate impact of ART on *S*1. Because the *S*1-infected population has a higher turnover, the effect of reducing the number of onward transmissions is seen more immediately in this population. The magnitude of the short-term ART effect is smaller than at equilibrium and is greatest when low CD4 transmissions are of greater importance to the high virulence strain (*S*1). The results are again summarized in table 2.

### 2.7. Can we find data to test these model predictions?

Since the HIV epidemic is only decades old and widespread ART is relatively recent, we expect that only the short-term effects can be observed at present. Inferring the ART effect from data from a single epidemic is not feasible. Instead we require comparable data from two similar epidemics that differ in their ART use (though it will not be possible to ensure that all other differences are negligible). Previously published data of this sort were introduced at the beginning of the results (figure 1*b*). VRC, an *in vitro* measure of viral fitness, was found to be significantly lower in data from Botswana than in South Africa, where the epidemic is ≈10 years younger. ART had been introduced ≈5 years previously in Botswana but was very limited in South Africa at the time the data were collected. This would suggest a decrease in virulence over time in the presence of ART, as we would expect from our model predictions. However, it is not possible to conclude that ART was a causal effector in that decrease.

## 3. Discussion

Several large observational studies, each spanning of the order of 20 years and using various markers of virulence, have been conducted with the aim of determining whether the virulence of HIV is changing, and some have found significant trends [3]. Interpretation of these results is hampered in part by the various measurement and sampling biases that could cause spurious effects, as discussed in detail elsewhere [3,37]. Additionally, there is perhaps an underlying assumption in the field that patterns of virulence change in a particular epidemic will be monotonic. By contrast, our model shows that the usual course of an epidemic and differing dynamics of the spread of high and low virulence viruses are likely to result in non-monotonic, transient changes in virulence. In particular, it is possible that observed decreases in virulence a few decades into an epidemic would be caused by the steadier spread of low virulence viruses and hence not necessarily a sign that the virus will eventually evolve to avirulence. Non-monotonic changes in mean SPVL have also been observed in agent-based model simulations [37], and very similar patterns to those described by our model have recently been seen in data pooled from seroconverter cohorts across Europe [29]. Measurements of SPVL in over 15 000 individuals with estimated seroconversion dates ranging from 1979 to 2008 showed patterns of increasing SPVL from 1980 to 2002 followed by a tendency of returning to lower levels in recent years. The global HIV-1 pandemic is composed of several sub-epidemics which differ in terms of the age of the epidemic, viral subtype, mode of transmission, population structure and virulence of the virus seeding the epidemic, among other factors. Therefore, given that our model shows non-monotonic patterns of virulence change we are not surprised that studies on different HIV-1-infected populations have found differing trends.

Our intuition was that treatment of the most sick patients would favour the less virulent viruses and that over time this might drive lower and lower average virulence. However, analysis with our PDE model suggested that we may better understand the effect of ART on virulence when we think in terms of the relative importance (to each strain) of ‘low CD4’ transmissions, i.e. the transmissions that no longer occur when a patient is on ART. We then see that the effects expected by our intuition could in theory come about. For example, low CD4 transmissions are of greater importance to the high virulence viruses if patients with low CD4 counts progress at the same rate, independent of SPVL. However, it seems likely that ‘low CD4’ transmissions are of similar importance to both strains, and hence the resulting ART effect may not be significant. In practice, we are also unlikely to observe a large effect of ART on virulence since these long-term effects materialize over the timescale of centuries and the largest effects also require high coverage of fully suppressive ART. Taking the epidemic in Africa as an example, ART coverage in the WHO African region was reported to be 37% in 2013 [17]. Yet, given that the HIV-1 epidemic is far from over in spite of substantial ART coverage [20], it is important to understand the size and direction of any putative ART effect.

We purposefully used an over-simplified model for the main analyses, because it allowed us to gain an understanding of the key aspects of the dynamics. However, it is instructive to consider further the following possibilities that were not initially allowed for: (i) low heritability of SPVL; (ii) varying infectiousness during the course of untreated infection and continued transmission in patients on treatment; (iii) heterogeneity in contact rates; and (iv) early ART initiation, independent of CD4 count.

Firstly, we have assumed that the SPVL of a new infection is completely determined by the viral strain, and kept the strain switching rate, *m*, low. Consequently, we have modelled a situation where heritability of SPVL is high and there is strong selection for virulence at the between-host level. The debate surrounding these two issues is still ongoing (see [7,8,16,38,39] for further detail). If there is no possibility of virulence adaptation in response to between-host selection pressures then our analysis, while valid, is redundant. However, ART could still have an effect on virulence evolution that is shaped by within-host selection pressures because the introduction of ART may increase the proportion of transmissions occurring earlier in infection. The other possibility is that there is selection for virulence at the between-host level but that heritability of SPVL is low (in other words, the percentage of the variation in SPVL that is explained by the viral strain is low). For example, heritability of SPVL across sub-Saharan Africa has been estimated at 20–46% [7]. We adapted our model to account for this—see electronic supplementary material, §2 and figures S1–S3. Reducing the heritability brings the two extremes of equilibrium strain balance closer together and so the magnitude of the ART effect is reduced, both in the short term and long term, while the direction is largely unchanged.

Secondly, we have assumed that the probability of transmission is constant throughout infection. In reality, the proportion of transmissions occurring in acute infection could be elevated as a result of higher infectiousness and also depending on underlying sexual mixing patterns. In the electronic supplementary material (§3, figures S4–S6), the analyses are re-done under the assumption that 50% of onward transmissions occur in the first six months of infection, an approximate upper bound informed by the most recent analyses [14]. We have also made the assumption that patients in receipt of ART never transmit the virus again. This is optimistic given that continuous suppression of viral replication requires good adherence to medication that must be taken daily [40,41]. Allowing some individuals in the model to continue to transmit the virus at a lower rate would have effects akin to reducing the coverage of ART. Therefore, we have not presented separate analyses with this assumption relaxed.

Thirdly, it is known that differing modes of transmission or social factors influencing contact networks can have an effect on the progress of an epidemic [15]. We have assumed homogeneity of contacts in our model, whereas there may be situations in which a subset of the population (the ‘core’) has much higher contact rates. We may imagine that such a situation would result in differing patterns of virulence evolution in the core and in the ‘periphery’. However, adding this core/periphery structure to our model has little effect on the overall patterns of virulence evolution seen both with and without ART (electronic supplementary material, §4, figures S7–S8).

Fourthly and finally, in the light of recent evidence that treating early might reduce the size of the latent HIV reservoir and could possibly result in post-treatment control [42], some have suggested that all patients be treated as early as possible. Would we have seen a different pattern if we had modelled a set of treatment guidelines where patients are treated as soon as possible after diagnosis, regardless of CD4 count? Incorporating this method of treatment into our model, we see that it always favours the virulent strain in the long term (i.e. at equilibrium; electronic supplementary material, figure S9). However, in the short term the effect of ART is small at realistic levels of coverage (electronic supplementary material, figure S10).

Electronic supplementary material, table 1 summarizes the effect of all the additional considerations and modifications above relative to the main results of the original model. In almost all cases, the modifications result in a less severe ART effect, except when treatment efforts are concentrated towards those with more contacts. Indeed, as the relative importance of ‘low CD4’ transmissions has a tendency to be similar for both strains, it is hard to imagine a scenario or reasonable parameter regime that would result in drastically higher variations in virulence driven by treatment based on CD4 count. The results of a very recent independent study corroborate our main findings on the size and direction of the ART effect using different methods [43].

In conclusion, should the results of this study alter how we treat? We believe not. Note that we may never be able to answer questions of how much impact ART has had on the shape of virulence evolution in a particular epidemic. Because we will always be restricted to observational studies of virulence and no two epidemics differ only in ART coverage, we will not be able to use these data to establish causality. However, we have been able to use our model to look at the range of feasible dynamics of the epidemic with varying coverage of ART. The intuition that ART initiation based on CD4 count would benefit less virulent viruses is correct when ‘low CD4’ transmissions bear greater importance for the spread of the higher virulence strains. But, though there are scenarios in which ART might cause a substantial effect in the long run, the effects on the timescales that we are concerned with are small. Finally, we emphasize that we believe the effects of ART on virulence are far outweighed by the benefits of treatment both to the individuals being treated and in terms of halting onward transmission to others [44,45].

## Supplementary Material

Supplementary methods, additional analyses and figures accompanying these analyses
