# Supplementary material for: The impact of antiretroviral therapy on population-level virulence evolution of HIV-1
Source: J R Soc Interface. 2015 Dec 6;12(113):20150888. doi: 10.1098/rsif.2015.0888 (PMC4707861; doi:10.1098/rsif.2015.0888)
Supplement: Supplementary methods, additional analyses and figures accompanying these analyses [file rsif20150888supp1.pdf]

The Impact of Antiretroviral Therapy on Population Level Virulence Evolution of HIV-1

H. E. Roberts*, P. J. R. Goulder, A. R. McLean

*email: hannah.roberts@ndm.ox.ac.uk

1 Supplementary methods relating to the original model

In this section we cover in more detail the calculations using the PDE model that help us to understand when ART is expected to favour the less virulent strain. We then relate the PDE model to the ODE model at equilibrium and solve the full ODE model at equilibrium. This is done in order to calculate 'total number of AIDS-related deaths per untreated infected person per year' at equilibrium and its relation to the relative importance of low CD4 transmissions.

1.1 ODE model equations

Recall the equations for the ODE model with ART as presented in (2) in the main text. S represents the population of susceptible (uninfected) individuals, I are the various infected classes, where the superscripts H and L indicate high and low (resp.) SPVL and the subscripts S1 and S2 index strain type, with u and t distinguishing between individuals who will remain untreated or will be treated with ART. To shorten notation we denote $I_i^j = I_{i,t}^j + I_{i,u}^j$ and define $\lambda_i = (\beta_H c I_i^H + \beta_L c I_i^L)/N$. T is the population of individuals receiving ART, assumed to be noninfectious.

$$
\begin{aligned}
\frac{dS}{dt} &= B - (\lambda_{S1} + \lambda_{S2})S - \mu S \\
\frac{dI_{S1,u}^H}{dt} &= (1-k)\lambda_{S1}S - (\mu + a_H)I_{S1,u}^H - mI_{S1,u}^H + mI_{S2,u}^H \\
\frac{dI_{S1,t}^H}{dt} &= k\lambda_{S1}S - (\mu + p_H)I_{S1,t}^H - mI_{S1,t}^H + mI_{S2,t}^H \\
\frac{dI_{S1,u}^L}{dt} &= -(\mu + a_L)I_{S1,u}^L - mI_{S1,u}^L + mI_{S2,u}^L \\
\frac{dI_{S1,t}^L}{dt} &= -(\mu + p_L)I_{S1,t}^L - mI_{S1,t}^L + mI_{S2,t}^L \\
\frac{dI_{S2,u}^H}{dt} &= -(\mu + a_H)I_{S2,u}^H - mI_{S2,u}^H + mI_{S1,u}^H \\
\frac{dI_{S2,t}^H}{dt} &= -(\mu + p_H)I_{S2,t}^H - mI_{S2,t}^H + mI_{S1,t}^H \\
\frac{dI_{S2,u}^L}{dt} &= (1-k)\lambda_{S2}S - (\mu + a_L)I_{S2,u}^L - mI_{S2,u}^L + mI_{S1,u}^L \\
\frac{dI_{S2,t}^L}{dt} &= k\lambda_{S2}S - (\mu + p_L)I_{S2,t}^L - mI_{S2,t}^L + mI_{S1,t}^L \\
\frac{dT}{dt} &= p_H(I_{S1,t}^H + I_{S2,t}^H) + p_L(I_{S1,t}^L + I_{S2,t}^L) - \mu T
\end{aligned}
\tag{1}
$$

1.2 PDE model definition and solution at epidemic equilibrium

To recap, in the PDE model both treatment and AIDS-related death are dependent on age of infection. Therefore each infected class is no longer split into 'treated' and 'untreated'. Let τ denote time since infection, and define $y_1^H(t,\tau)$ to be the number of high SPVL virulent strain (S1) infections of infection-age τ at time t, so that

$$\int_0^\infty y_{S1}^H(t,\tau)\mathrm{d}\tau = I_{S1}^H(t)$$

and similarly for y_{S1}^L, y_{S2}^H, y_{S2}^L.

Let an average person with a high SPVL infection become classified as 'low CD4' at $\tau = T_H$ and an average person with a low SPVL infection become classified as 'low CD4' at $\tau = T_L$. Let d_H and d_L be the AIDS-related death rates of patients with high and low SPVL infections respectively. Then the full set of PDEs describing the spread of the two strains is:

$$\begin{aligned}
\frac{dS}{dt} &= B - (\lambda_{S1} + \lambda_{S2})S - \mu S \\
\frac{\partial y_{S1}^H}{\partial t} + \frac{\partial y_{S1}^H}{\partial \tau} &= \begin{cases} -(\mu + m)y_{S1}^H(t,\tau) + my_{S2}^H(t,\tau) & \text{if } \tau \le T_H \\ -(\mu + d_H + m)y_{S1}^H(t,\tau) + my_{S2}^H(t,\tau) & \text{if } \tau > T_H \end{cases} \qquad y_{S1}^H(t,0) = \lambda_{S1}S \\
\frac{\partial y_{S1}^L}{\partial t} + \frac{\partial y_{S1}^L}{\partial \tau} &= \begin{cases} -(\mu + m)y_{S1}^L(t,\tau) + my_{S2}^L(t,\tau) & \text{if } \tau \le T_L \\ -(\mu + d_L + m)y_{S1}^L(t,\tau) + my_{S2}^L(t,\tau) & \text{if } \tau > T_L \end{cases} \qquad y_{S1}^L(t,0) = 0 \\
\frac{\partial y_{S2}^H}{\partial t} + \frac{\partial y_{S2}^H}{\partial \tau} &= \begin{cases} -(\mu + m)y_{S2}^H(t,\tau) + my_{S1}^H(t,\tau) & \text{if } \tau \le T_H \\ -(\mu + d_H + m)y_{S2}^H(t,\tau) + my_{S1}^H(t,\tau) & \text{if } \tau > T_H \end{cases} \qquad y_{S2}^H(t,0) = 0 \\
\frac{\partial y_{S2}^L}{\partial t} + \frac{\partial y_{S2}^L}{\partial \tau} &= \begin{cases} -(\mu + m)y_{S2}^L(t,\tau) + my_{S1}^L(t,\tau) & \text{if } \tau \le T_L \\ -(\mu + d_L + m)y_{S2}^L(t,\tau) + my_{S1}^L(t,\tau) & \text{if } \tau > T_L \end{cases} \qquad y_{S2}^L(t,0) = \lambda_{S2}S
\end{aligned}$$

where $\lambda_{S1} = (\beta_H c I_H^{S1} + \beta_L c I_L^{S1})/N$ and $\lambda_{S2} = (\beta_H c I_H^{S2} + \beta_L c I_L^{S2})/N$. There is also a discontinuity at $\tau = T_i$ where a proportion k of infected patients reaching that infected age are put onto treatment (and hence removed from the infectious class):

$$\lim_{\tau \to T_j^+} y_i^j(\tau) = (1-k) \lim_{\tau \to T_j^-} y_i^j(\tau)$$

At equilibrium, when $\frac{\partial y_i^j}{\partial t} = 0$, the above reduce to a set of ODEs in τ:

$$\frac{\mathrm{d}y_{S1}^H}{\mathrm{d}\tau} = \begin{cases} -(\mu+m)y_{S1}^H(\tau) + my_{S2}^H(\tau) & \text{if } \tau \le T_H \\ -(\mu+d_H+m)y_{S1}^H(\tau) + my_{S2}^H(\tau) & \text{if } \tau > T_H \end{cases} \qquad y_{S1}^H(0) = \lambda_{S1}S^*$$

$$\frac{\mathrm{d}y_{S1}^L}{\mathrm{d}\tau} = \begin{cases} -(\mu+m)y_{S1}^L(\tau) + my_{S2}^L(\tau) & \text{if } \tau \le T_L \\ -(\mu+d_L+m)y_{S1}^L(\tau) + my_{S2}^L(\tau) & \text{if } \tau > T_L \end{cases} \qquad y_{S1}^L(0) = 0$$

$$\frac{\mathrm{d}y_{S2}^H}{\mathrm{d}\tau} = \begin{cases} -(\mu+m)y_{S2}^H(\tau) + my_{S1}^H(\tau) & \text{if } \tau \le T_H \\ -(\mu+d_H+m)y_{S2}^H(\tau) + my_{S1}^H(\tau) & \text{if } \tau > T_H \end{cases} \qquad y_{S2}^H(0) = 0$$

$$\frac{\mathrm{d}y_{S2}^L}{\mathrm{d}\tau} = \begin{cases} -(\mu+m)y_{S2}^L(\tau) + my_{S1}^L(\tau) & \text{if } \tau \le T_L \\ -(\mu+d_L+m)y_{S2}^L(\tau) + my_{S1}^L(\tau) & \text{if } \tau > T_L \end{cases} \qquad y_{S2}^L(0) = \lambda_{S2}S^*$$

where S^* is the equilibrium value of S and so $\lambda_{S1}S^*, \lambda_{S2}S^*$ are now constants (independent of τ). We may solve these for $y_{S1}^j + y_{S2}^j$ and $y_{S1}^j - y_{S2}^j$:

$$y_{S1}^H + y_{S2}^H = \begin{cases} \lambda_{S1}S^* e^{-\mu\tau} & \text{if } \tau \le T_H \\ (1-k)\lambda_{S1}S e^{-\mu T_H} e^{-(\mu+d_H)(\tau-T_H)} & \text{if } \tau > T_H \end{cases}$$

$$y_{S1}^H - y_{S2}^H = \begin{cases} \lambda_{S1}S^* e^{-(\mu+2m)\tau} & \text{if } \tau \le T_H \\ (1-k)\lambda_{S1}S e^{-(\mu+2m)T_H} e^{-(\mu+2m+d_H)(\tau-T_H)} & \text{if } \tau > T_H \end{cases}$$

$$y_{S1}^L + y_{S2}^L = \begin{cases} \lambda_{S2}S^* e^{-\mu\tau} & \text{if } \tau \le T_L \\ (1-k)\lambda_{S2}S e^{-\mu T_L} e^{-(\mu+d_L)(\tau-T_L)} & \text{if } \tau > T_L \end{cases}$$

$$y_{S1}^L - y_{S2}^L = -\begin{cases} \lambda_{S2}S^* e^{-(\mu+2m)\tau} & \text{if } \tau \le T_L \\ (1-k)\lambda_{S2}S e^{-(\mu+2m)T_L} e^{-(\mu+2m+d_L)(\tau-T_L)} & \text{if } \tau > T_L \end{cases}$$

Then

$$\begin{aligned} 2y_{S1}^H &= \lambda_{S1}S^*[e^{-\mu\tau} + e^{-(\mu+2m)\tau}] \times \begin{cases} 1 & \text{if } \tau \le T_H \\ (1-k)e^{-d_H(\tau-T_H)} & \text{if } \tau > T_H \end{cases} \\ 2y_{S2}^H &= \lambda_{S1}S^*[e^{-\mu\tau} - e^{-(\mu+2m)\tau}] \times \begin{cases} 1 & \text{if } \tau \le T_H \\ (1-k)e^{-d_H(\tau-T_H)} & \text{if } \tau > T_H \end{cases} \\ 2y_{S1}^L &= \lambda_{S2}S^*[e^{-\mu\tau} - e^{-(\mu+2m)\tau}] \times \begin{cases} 1 & \text{if } \tau \le T_L \\ (1-k)e^{-d_L(\tau-T_L)} & \text{if } \tau > T_L \end{cases} \\ 2y_{S2}^L &= \lambda_{S2}S^*[e^{-\mu\tau} + e^{-(\mu+2m)\tau}] \times \begin{cases} 1 & \text{if } \tau \le T_L \\ (1-k)e^{-d_L(\tau-T_L)} & \text{if } \tau > T_L \end{cases} \end{aligned} \qquad (2)$$

Recall that the purpose of introducing age-of-infection dependent death rates and treatment was to determine for which parameter sets the following inequality is true

$$\Pr(\text{infected with strain S1} \mid \text{low CD4}) > \Pr(\text{infected with strain S1})$$

Here the sample space is the population of currently infected individuals and we are concerned with how these probabilities compare in the absence of ART ($k = 0$). This can be written as:

$$\frac{\int_{T_H}^{\infty} y_{S1}^H \mathrm{d}\tau + \int_{T_L}^{\infty} y_{S1}^L \mathrm{d}\tau}{\int_{T_H}^{\infty} y_{S1}^H + y_{S2}^H \mathrm{d}\tau + \int_{T_L}^{\infty} y_{S1}^L + y_{S2}^L \mathrm{d}\tau} > \frac{\int_{0}^{\infty} y_{S1}^H \mathrm{d}\tau + \int_{0}^{\infty} y_{S1}^L \mathrm{d}\tau}{\int_{0}^{\infty} y_{S1}^H + y_{S2}^H \mathrm{d}\tau + \int_{0}^{\infty} y_{S1}^L + y_{S2}^L \mathrm{d}\tau} \tag{3}$$

We may now use the expressions in (3) for $y_{S1}^H(\tau)$ etc., with $k = 0$, to calculate both sides of (4) at equilibrium. First the left hand side:

$$\begin{aligned}
\int_{T_H}^{\infty} y_{S1}^H \mathrm{d}\tau &= \frac{1}{2}\lambda_{S1} S^* \int_{T_H}^{\infty} \left(e^{-\mu\tau} + e^{-(\mu+2m)\tau}\right) e^{-a_h(\tau - T_H)} \mathrm{d}\tau \\
&= \frac{1}{2}\lambda_{S1} S^* e^{d_H T_H} \int_{T_H}^{\infty} e^{-(\mu+d_H)\tau} + e^{-(\mu+2m+d_H)\tau} \mathrm{d}\tau \\
&= \frac{1}{2}\lambda_{S1} S^* \left[-\frac{e^{-(\mu+d_H)\tau}}{\mu+d_H} - \frac{e^{-(\mu+2m+d_H)\tau}}{\mu+2m+d_H}\right]_{T_H}^{\infty} \\
&= \frac{1}{2}\lambda_{S1} S^* \left[\frac{e^{-(\mu+d_H)T_H}}{\mu+d_H} + \frac{e^{-(\mu+2m+d_H)T_H}}{\mu+2m+d_H}\right] \\
&= \frac{1}{2}\lambda_{S1} S^* \left(\frac{e^{-\mu T_H}}{\mu+d_H} + \frac{e^{-(\mu+2m)T_H}}{\mu+2m+d_H}\right)
\end{aligned}$$

Similarly

$$\begin{aligned}
\int_{T_H}^{\infty} y_{S2}^H \mathrm{d}\tau &= \frac{1}{2}\lambda_{S1} S^* \left(\frac{e^{-\mu T_H}}{\mu+d_H} - \frac{e^{-(\mu+2m)T_H}}{\mu+2m+d_H}\right) \\
\int_{T_L}^{\infty} y_{S1}^L \mathrm{d}\tau &= \frac{1}{2}\lambda_{S2} S^* \left(\frac{e^{-\mu T_L}}{\mu+d_L} - \frac{e^{-(\mu+2m)T_L}}{\mu+2m+d_L}\right) \\
\int_{T_L}^{\infty} y_{S2}^L \mathrm{d}\tau &= \frac{1}{2}\lambda_{S2} S^* \left(\frac{e^{-\mu T_L}}{\mu+d_L} + \frac{e^{-(\mu+2m)T_L}}{\mu+2m+d_L}\right)
\end{aligned}$$

Therefore

$$\int_{T_H}^{\infty} y_{S1}^H + y_{S2}^H \mathrm{d}\tau + \int_{T_L}^{\infty} y_{S1}^L + y_{S2}^L \mathrm{d}\tau = \lambda_{S1} S^* \frac{e^{-\mu T_H}}{\mu+d_H} + \lambda_{S2} S^* \frac{e^{-\mu T_L}}{\mu+d_L}$$

Also, for the right hand side

$$\begin{aligned}
\int_{0}^{\infty} y_{S1}^H \mathrm{d}\tau &= \int_{T_H}^{\infty} y_{S1}^H \mathrm{d}\tau + \int_{0}^{T_H} y_{S1}^H \mathrm{d}\tau \\
&= \int_{T_H}^{\infty} y_{S1}^H \mathrm{d}\tau + \frac{1}{1}\lambda_{S1} S^* \left[-\frac{e^{-\mu\tau}}{\mu} - \frac{e^{-(\mu+2m)\tau}}{\mu+2m}\right]_0^{T_H} \\
&= \int_{T_H}^{\infty} y_{S1}^H \mathrm{d}\tau + \frac{1}{2}\lambda_{S1} S^* \left[\frac{1}{\mu} + \frac{1}{\mu+2m} - \frac{e^{-\mu T_H}}{\mu} - \frac{e^{-(\mu+2m)T_H}}{\mu+2m}\right] \\
&= \frac{1}{2}\lambda_{S1} S^* \left[\frac{1}{\mu} + \frac{1}{\mu+2m} + e^{-\mu T_H}\left(\frac{1}{\mu+d_H} - \frac{1}{\mu}\right) + e^{-(\mu+2m)T_H}\left(\frac{1}{\mu+2m+d_H} - \frac{1}{\mu+2m}\right)\right]
\end{aligned}$$

and similarly to before the integral of all four populations is:

$$\lambda_{S1}S^*\left[\frac{1}{\mu}+e^{-\mu T_H}\left(\frac{1}{\mu+d_H}-\frac{1}{\mu}\right)\right]+\lambda_{S2}S^*\left[\frac{1}{\mu}+e^{-\mu T_L}\left(\frac{1}{\mu+d_L}-\frac{1}{\mu}\right)\right]$$

Substituting these all into equation 3, cross multiplying and comparing the $\lambda_{S1}\lambda_{S1}$, $\lambda_{S1}\lambda_{S2}$ and $\lambda_{S2}\lambda_{S2}$ terms on both sides we obtain

$$\begin{aligned}&\frac{1}{\mu}\left[\left(\lambda_{S1}+\lambda_{S2}-\lambda_{S1}e^{-\mu T_H}-\lambda_{S2}e^{-\mu T_L}\right)\left(\lambda_{S1}\frac{e^{-(\mu+2m)T_H}}{\mu+2m+d_H}-\lambda_{S2}\frac{e^{-(\mu+2m)T_L}}{\mu+2m+d_L}\right)\right]\\ &>\frac{1}{\mu+2m}\left[\left(\lambda_{S1}\frac{e^{-\mu T_H}}{\mu+d_H}+\lambda_{S2}\frac{e^{-\mu T_L}}{\mu+d_L}\right)\left(\lambda_{S2}e^{-(\mu+2m)T_L}-\lambda_{S1}e^{-(\mu+2m)T_H}+\lambda_{S1}-\lambda_{S2}\right)\right]\end{aligned}$$

To simplify further we set $m=0$, which is reasonable when m is very small compared to μ. So, with this assumption we are able to obtain

$$\begin{aligned}&\left[\left(\lambda_{S1}+\lambda_{S2}-\lambda_{S1}e^{-\mu T_H}-\lambda_{S2}e^{-\mu T_L}\right)\left(\lambda_{S1}\frac{e^{-\mu T_H}}{\mu+d_H}-\lambda_{S2}\frac{e^{-\mu T_L}}{\mu+d_L}\right)\right]\\ &\quad>\left[\left(\lambda_{S1}\frac{e^{-\mu T_H}}{\mu+d_H}+\lambda_{S2}\frac{e^{-\mu T_L}}{\mu+d_L}\right)\left(\lambda_{S2}e^{-\mu T_L}-\lambda_{S1}e^{-\mu T_H}+\lambda_{S1}-\lambda_{S2}\right)\right]\\ \Rightarrow\quad&2\lambda_{S2}\lambda_{S1}\frac{e^{-\mu T_H}}{\mu+d_H}-2\lambda_{S1}\lambda_{S2}\frac{e^{-\mu T_L}}{\mu+d_L}>2\lambda_{S1}\lambda_{S2}\frac{e^{-\mu T_L}e^{-\mu T_H}}{\mu+d_H}-2\lambda_{S1}\lambda_{S2}\frac{e^{-\mu T_H}e^{-\mu T_L}}{\mu+d_L}\\ \Rightarrow\quad&\frac{e^{\mu T_L}}{\mu+d_H}-\frac{e^{\mu T_H}}{\mu+d_L}>\frac{1}{\mu+d_H}-\frac{1}{\mu+d_L}\\ \Rightarrow\quad&\frac{e^{\mu T_L}-1}{e^{\mu T_H}-1}>\frac{\mu+d_H}{\mu+d_L}\end{aligned}$$

1.3 Relating the ODE and PDE models at equilibrium

As covered briefly in the main text, we wish to relate the parameters a_j and p_j in the ODE model to the parameters T_j and d_j in the PDE model. We do this by equating the two models at equilibrium, i.e. ensuring that at equilibrium the sizes of both the high SPVL and low SPVL infected classes in the ODE model match those in the PDE model. So, for $j=H,L$:

$$\begin{aligned}&\int_0^\infty y^j(t,\tau)\mathrm{d}\tau=I_u^j+I_t^j\\ \Leftrightarrow\quad&\int_0^{T_j}\lambda S^*e^{-\mu\tau}\mathrm{d}\tau\\ &+\int_{T_j}^\infty(1-k)\lambda S^*e^{-\mu T_j}e^{-(\mu+d_j)(\tau-T_j)}\mathrm{d}\tau=\frac{(1-k)\lambda S^*}{\mu+a_j}+\frac{k\lambda S^*}{\mu+p_j}\\ \Leftrightarrow\quad&\frac{\lambda S^*}{\mu}-\frac{\lambda S^*}{\mu}e^{-\mu T_j}+\frac{(1-k)\lambda S^*}{\mu+d_j}e^{-\mu T_j}=\frac{(1-k)\lambda S^*}{\mu+a_j}+\frac{k\lambda S^*}{\mu+p_j}\\ \Leftrightarrow\quad&e^{-\mu T_j}\left[\frac{1-k}{\mu+d_j}-\frac{1}{\mu}\right]=\frac{(1-k)}{\mu+a_j}+\frac{k}{\mu+p_j}+\frac{1}{\mu}\end{aligned}$$

Substituting in $k = 0$ and $k = 1$ shows that in order to obtain a condition that is independent of k we must have $\mu + d_j = p_j(\mu + a_j)/(p_j - a_j)$. Substituting this back in:

$$\begin{aligned}
& e^{-\mu T_j}\left[\frac{(1-k)(p_j - a_j)}{p_i(\mu + a_j)} - \frac{1}{\mu}\right] = \frac{1-k}{\mu + a_j} + \frac{k}{\mu + p_j} - \frac{1}{\mu} \\
\Leftrightarrow \quad & e^{-\mu T_j}\left[-\frac{\mu(kp_j + (1-k)a_j) + a_j p_j}{\mu p_j(\mu + a_j)}\right] = -\frac{\mu(kp_j + (1-k)a_j) + a_j p_j}{(\mu + a_j)\mu(\mu + p_j)} \\
\Leftrightarrow \quad & e^{-\mu T_j} = \frac{p_j}{\mu + p_j}
\end{aligned}$$

So we must choose a_i and p_j to satisfy

$$e^{-\mu T_j} = \frac{p_j}{\mu + p_j} \quad \text{and} \quad d_j = \frac{a_j(\mu + p_j)}{p_j - a_j}$$

1.4 Equilibrium population size calculations for the ODE model

To find the equilibrium population class sizes we first re-write the model equations in (4) in matrix notation. Let $\mathbf{dy}$ be the vector of derivatives, ordered as in (4). Similarly let $\mathbf{y}$ be the vector of population sizes. Then define the matrices $\mathbf{\Lambda}$ and $\mathbf{M}$ such that

$$\mathbf{dy} = \mathbf{\Lambda y} S/N + \mathbf{My}$$

At equilibrium $\mathbf{dy} = \mathbf{0}$ and so we wish to solve $\mathbf{\Lambda y} S^*/N^* = -\mathbf{My}$, where S^* and N^* are the equilibrium values of S and N. The solution for $\mathbf{y}$ that we want is the eigenvector of $\mathbf{M^{-1}\Lambda}$ corresponding to the eigenvalue with the largest absolute value.

Now let $\delta_1 = \mu + a_H + m$, $\delta_2 = \mu + p_H + m$, $\delta_3 = \mu + a_L + m$, $\delta_4 = \mu + p_L + m$ so that

$$\mathbf{M} = \begin{pmatrix}
-\delta_1 & 0 & 0 & 0 & m & 0 & 0 & 0 \\
0 & -\delta_2 & 0 & 0 & 0 & m & 0 & 0 \\
0 & 0 & -\delta_3 & 0 & 0 & 0 & m & 0 \\
0 & 0 & 0 & -\delta_4 & 0 & 0 & 0 & m \\
m & 0 & 0 & 0 & -\delta_1 & 0 & 0 & 0 \\
0 & m & 0 & 0 & 0 & -\delta_2 & 0 & 0 \\
0 & 0 & m & 0 & 0 & 0 & -\delta_3 & 0 \\
0 & 0 & 0 & m & 0 & 0 & 0 & -\delta_4
\end{pmatrix}$$

and we can calculate the inverse

$$\mathbf{M^{-1}} = \begin{pmatrix}
m_1 & 0 & 0 & 0 & n_1 & 0 & 0 & 0 \\
0 & m_2 & 0 & 0 & 0 & n_2 & 0 & 0 \\
0 & 0 & m_3 & 0 & 0 & 0 & n_3 & 0 \\
0 & 0 & 0 & m_4 & 0 & 0 & 0 & n_4 \\
n_1 & 0 & 0 & 0 & m_1 & 0 & 0 & 0 \\
0 & n_2 & 0 & 0 & 0 & m_2 & 0 & 0 \\
0 & 0 & n_3 & 0 & 0 & 0 & m_3 & 0 \\
0 & 0 & 0 & n_4 & 0 & 0 & 0 & m_4
\end{pmatrix}$$

where $m_i = \frac{\delta_i}{m^2-\delta_i^2}$, $n_i = \frac{m}{\delta_i^2-m^2}$ for $i \in \{1,2,3,4\}$. This gives

$$\mathbf{M^{-1}\Lambda} = c\begin{pmatrix} m_1\bar{k}\beta_H & m_1\bar{k}\beta_H & m_1\bar{k}\beta_L & m_1\bar{k}\beta_L & 0 & 0 & 0 & 0 \\ m_2k\beta_H & m_2k\beta_H & m_2k\beta_L & m_2k\beta_L & 0 & 0 & 0 & 0 \\ 0 & 0 & 0 & 0 & n_3\bar{k}\beta_H & n_3\bar{k}\beta_H & n_3\bar{k}\beta_L & n_3\bar{k}\beta_L \\ 0 & 0 & 0 & 0 & n_4k\beta_H & n_4k\beta_H & n_4k\beta_L & n_4k\beta_L \\ n_1\bar{k}\beta_H & n_1\bar{k}\beta_H & n_1\bar{k}\beta_L & n_1\bar{k}\beta_L & 0 & 0 & 0 & 0 \\ n_2k\beta_H & n_2k\beta_H & n_2k\beta_L & n_2k\beta_L & 0 & 0 & 0 & 0 \\ 0 & 0 & 0 & 0 & m_3\bar{k}\beta_H & m_3\bar{k}\beta_H & m_3\bar{k}\beta_L & m_3\bar{k}\beta_L \\ 0 & 0 & 0 & 0 & m_4k\beta_H & m_4k\beta_H & m_4k\beta_L & m_4k\beta_L \end{pmatrix}$$

where $\bar{k} = (1-k)$. We can calculate the eigenvalues and eigenvectors of this matrix computationally. If $\mathbf{y}^*$ is the eigenvector corresponding to the largest eigenvalue then the equilibrium value of total AIDS-related deaths per untreated infected person per year is $\sum_i (a_H y_{i,u}^{*H} + a_L y_{i,u}^{*L}) / \sum_i (y_{i,u}^{*H} + y_{i,u}^{*L})$, as plotted in figure 5 (main text).

2 Low heritability of SPVL

2.1 Methods

In this new model an infection initiated with strain S1 has high SPVL with frequency $f \in (0.5, 1)$ (whereas before $f = 1$) and remains at the same SPVL regardless of within host strain switching (as before). The same is true for infections initiated by strain S2 and low SPVL. The lower f is, the lower the heritability of SPVL in the model. The adjusted model equations are:

$$\begin{aligned}
\frac{dS}{dt} &= B - (\lambda_{S1} + \lambda_{S2})S - \mu S \\
\frac{dI^H_{S1,u}}{dt} &= f(1-k)\lambda_{S1}S - (\mu + a_H)I^H_{S1,u} - mI^H_{S1,u} + mI^H_{S2,u} \\
\frac{dI^H_{S1,t}}{dt} &= fk\lambda_{S1}S - (\mu + p_H)I^H_{S1,t} - mI^H_{S1,t} + mI^H_{S2,t} \\
\frac{dI^L_{S1,u}}{dt} &= (1-f)(1-k)\lambda_{S1}S - (\mu + a_L)I^L_{S1,u} - mI^L_{S1,u} + mI^L_{S2,u} \\
\frac{dI^L_{S1,t}}{dt} &= (1-f)k\lambda_{S1}S - (\mu + p_L)I^L_{S1,t} - mI^L_{S1,t} + mI^L_{S2,t} \\
\frac{dI^H_{S2,u}}{dt} &= (1-f)(1-k)\lambda_{S2}S - (\mu + a_H)I^H_{S2,u} - mI^H_{S2,u} + mI^H_{S1,u} \\
\frac{dI^H_{S2,t}}{dt} &= (1-f)k\lambda_{S2}S - (\mu + p_H)I^H_{S2,t} - mI^H_{S2,t} + mI^H_{S1,t} \\
\frac{dI^L_{S2,u}}{dt} &= f(1-k)\lambda_{S2}S - (\mu + a_L)I^L_{S2,u} - mI^L_{S2,u} + mI^L_{S1,u} \\
\frac{dI^L_{S2,t}}{dt} &= fk\lambda_{S2}S - (\mu + p_L)I^L_{S2,t} - mI^L_{S2,t} + mI^L_{S1,t} \\
\frac{dT}{dt} &= p_H(I^H_{S1,t} + I^H_{S2,t}) + p_L(I^L_{S1,t} + I^L_{S2,t}) - \mu T
\end{aligned} \tag{4}$$

Note that a low strain switching rate, m, is still required to avoid one strain necessarily becoming extinct.

The R_0 values are now calculated as (approximating using $m = 0$ again):

$$\begin{aligned}
R_0 = {} & \Pr(\text{new infection has high SPVL}) \times (\text{avg. \# infections generated by high SPVL infection}) \\
& + \Pr(\text{new infection has low SPVL}) \times (\text{avg. \# infections generated by low SPVL infection})
\end{aligned}$$

Hence:

$$\begin{aligned}
R_0^{S1} &= f\beta_H c\left(\frac{(1-k)}{\mu + a_H} + \frac{k}{\mu + p_H}\right) + (1-f)\beta_L c\left(\frac{(1-k)}{\mu + a_L} + \frac{k}{\mu + p_L}\right) \\
R_0^{S2} &= (1-f)\beta_H c\left(\frac{(1-k)}{\mu + a_H} + \frac{k}{\mu + p_H}\right) + f\beta_L c\left(\frac{(1-k)}{\mu + a_L} + \frac{k}{\mu + p_L}\right)
\end{aligned}$$

And the equilibrium matrix becomes:

$$\mathbf{M^{-1}\Lambda} = c\begin{pmatrix}
m_1 f\bar{k}\beta_H & m_1 f\bar{k}\beta_H & m_1 f\bar{k}\beta_L & m_1 f\bar{k}\beta_L & n_1\bar{f}\bar{k}\beta_H & n_1\bar{f}\bar{k}\beta_H & n_1\bar{f}\bar{k}\beta_L & n_1\bar{f}\bar{k}\beta_L \\
m_2 fk\beta_H & m_2 fk\beta_H & m_2 fk\beta_L & m_2 fk\beta_L & n_2\bar{f}k\beta_H & n_2\bar{f}k\beta_H & n_2\bar{f}k\beta_L & n_2\bar{f}k\beta_L \\
m_3\bar{f}\bar{k}\beta_H & m_3\bar{f}\bar{k}\beta_H & m_3\bar{f}\bar{k}\beta_L & m_3\bar{f}\bar{k}\beta_L & n_3 f\bar{k}\beta_H & n_3 f\bar{k}\beta_H & n_3 f\bar{k}\beta_L & n_3 f\bar{k}\beta_L \\
m_4\bar{f}k\beta_H & m_4\bar{f}k\beta_H & m_4\bar{f}k\beta_L & m_4\bar{f}k\beta_L & n_4 fk\beta_H & n_4 fk\beta_H & n_4 fk\beta_L & n_4 fk\beta_L \\
n_1 f\bar{k}\beta_H & n_1 f\bar{k}\beta_H & n_1 f\bar{k}\beta_L & n_1 f\bar{k}\beta_L & m_1\bar{f}\bar{k}\beta_H & m_1\bar{f}\bar{k}\beta_H & m_1\bar{f}\bar{k}\beta_L & m_1\bar{f}\bar{k}\beta_L \\
n_2 fk\beta_H & n_2 fk\beta_H & n_2 fk\beta_L & n_2 fk\beta_L & m_2\bar{f}k\beta_H & m_2\bar{f}k\beta_H & m_2\bar{f}k\beta_L & m_2\bar{f}k\beta_L \\
n_3\bar{f}\bar{k}\beta_H & n_3\bar{f}\bar{k}\beta_H & n_3\bar{f}\bar{k}\beta_L & n_3\bar{f}\bar{k}\beta_L & m_3 f\bar{k}\beta_H & m_3 f\bar{k}\beta_H & m_3 f\bar{k}\beta_L & m_3 f\bar{k}\beta_L \\
n_4\bar{f}k\beta_H & n_4\bar{f}k\beta_H & n_4\bar{f}k\beta_L & n_4\bar{f}k\beta_L & m_4 fk\beta_H & m_4 fk\beta_H & m_4 fk\beta_L & m_4 fk\beta_L
\end{pmatrix}$$

where $\bar{f} = (1 - f)$.

2.2 Results: Magnitude and direction of the ART effect

We have not re-done the PDE analysis for this model since we expect the direction of the ART-effect at equilibrium to remain the same as in the original model. This is because, even though high SPVL infections don't correspond exactly to strain S1 infections, it is still the case that if 'low CD4' transmissions occur proportionally more in high SPVL infections (compared to low SPVL infections) then they are of greater importance to the onward transmission of S1. Therefore, so long as $f > 0.5$, the direction of the long-term ART-effect will remain the same.

In supplementary figures 1-3 we have redone figures 3, 5 and 6 (resp.) of the main text using this model and $f = 0.8$. We see that, in both the short- and the long-term, the magnitude of the ART-effect on virulence is lessened. This is because reducing the heritability brings the number of high and low SPVL infections closer together, and so there is less scope for change in overall virulence. Aside from this the results are qualitatively the same; the figures show that the patterns of virulence change in the absence of ART and the direction of the ART-effect in the short- and long-term are all very similar to in the original model.

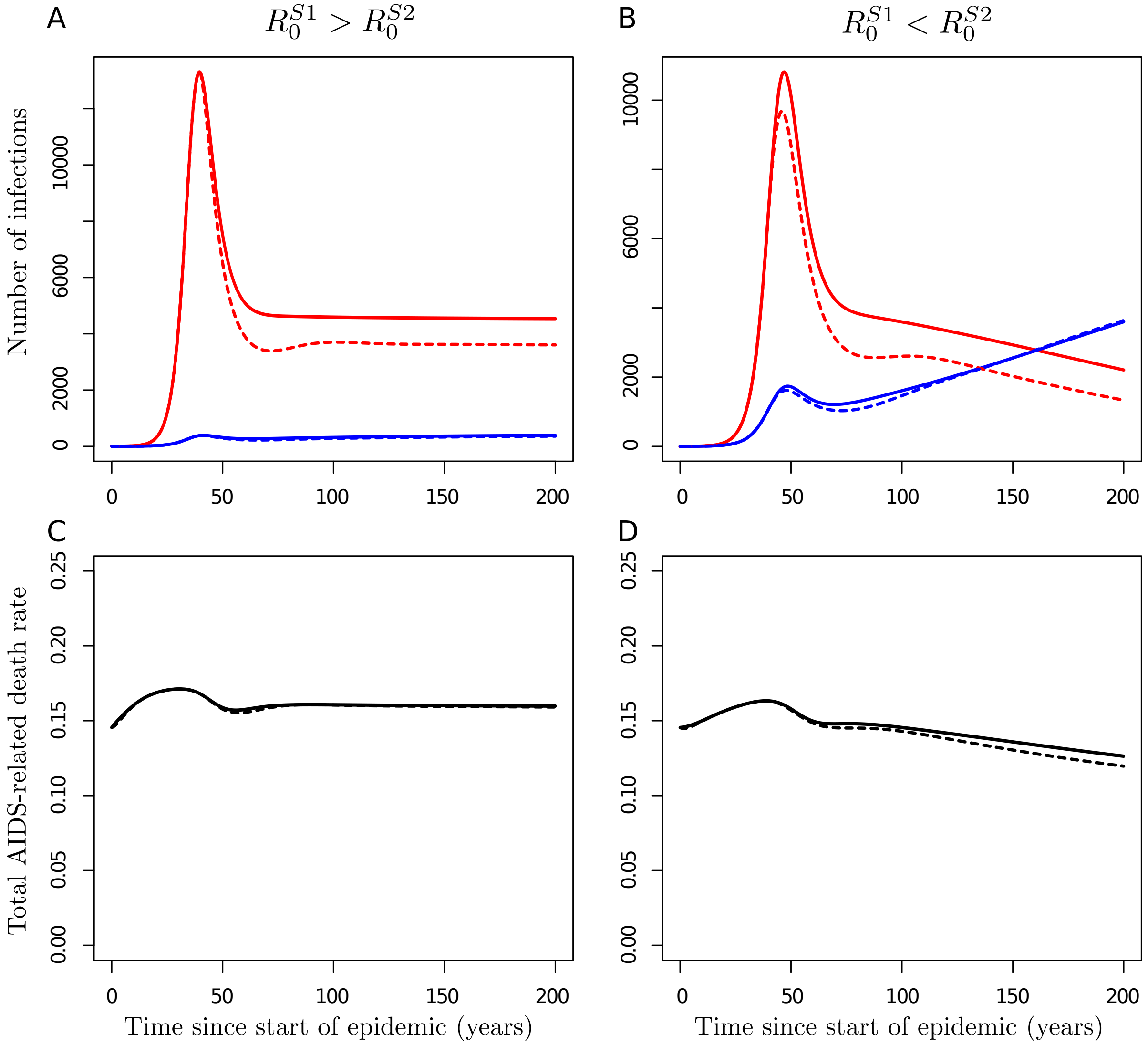

Figure S1:**Behaviour of the model with reduced heritability, with and without ART.** Parameters and initial conditions are as in figure 3 (main text) but with $f = 0.8$. A, C: In the absence of ART $R_0^{S1} = 2.4, R_0^{S2} = 2.2$. B, D: In the absence of ART $R_0^{S1} = 2.2, R_0^{S2} = 2.4$. Solid lines show the output of the model without ART while dotted lines plot trajectories for the model when ART is introduced at 30% coverage 40 years into the epidemic. A, B: The number of strain S1 infections through time is shown in red and the number of strain S2 infections in blue. With reduced heritability, the numbers of S1 and S2 infections are similar to in the case where $f = 1$ (figure 3, main text). C, D: However since each strain now gives rise to a mixture of high and low SPVL infections, the numbers of high and low SPVL infections are closer to each other and this results in the smaller magnitude variations in virulence. The shape of virulence change over time is similar to when $f = 1$. The effect of ART is still to increase virulence compared to the model without ART, but this effect is also reduced in magnitude when heritability is reduced.

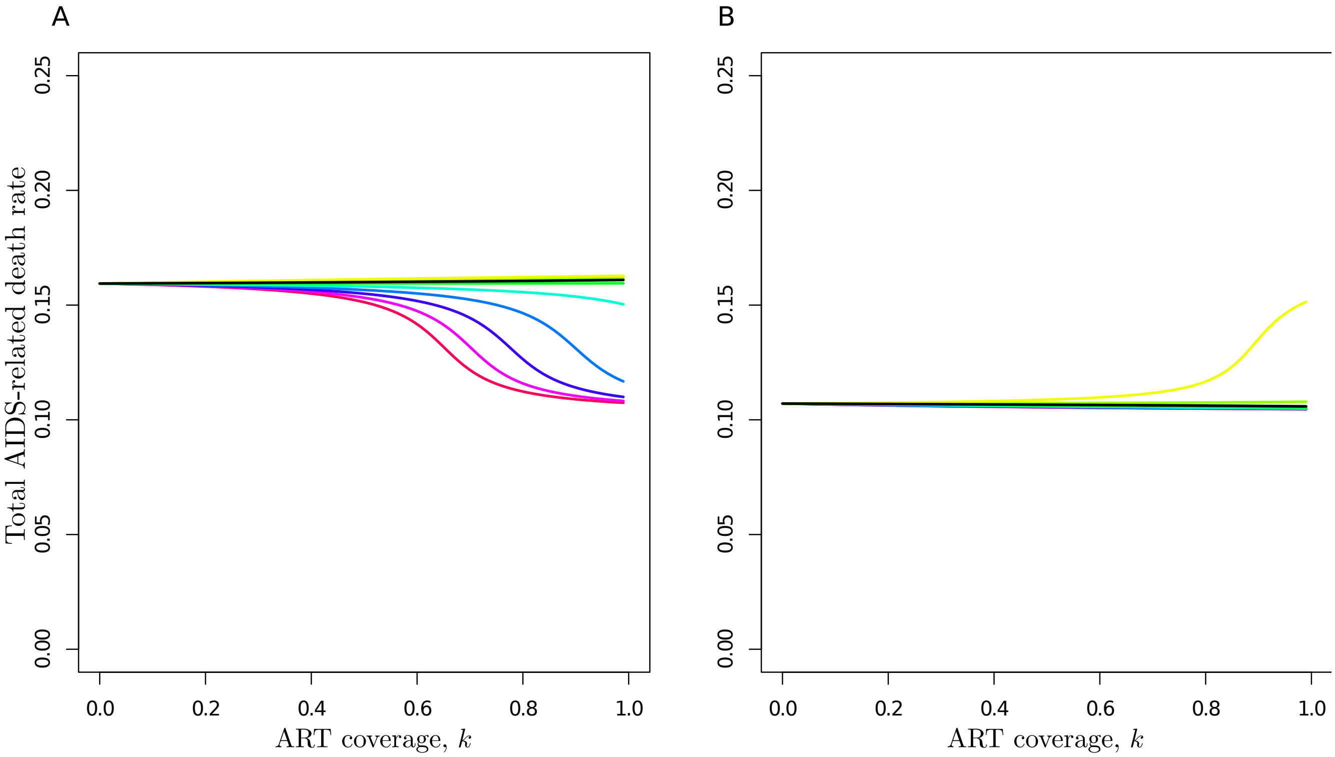

Figure S2: **The relationship between ART coverage and virulence at equilibrium for different parameter sets, when $f = 0.8$.** Parameters and colours as in figure 5 (main text) but with the heritability parameter $f = 0.8$. Recall $\frac{B_H}{A_H}$ is kept constant while $\frac{B_L}{A_L}$ is varied from low (red line) to high (yellow line). A: $R_0^{S1} = 2.4, R_0^{S2} = 2.2$. B: $R_0^{S1} = 2.2, R_0^{S2} = 2.4$. Total number of AIDS-related deaths per untreated infected person per year is plotted on the y axis. Since each strain now gives rise to a mixture of high and low virulence infections, the overall virulence values that occur when one strain dominates and the other is maintained at mutation-selection balance are less extreme than in figure 5 (main text) when $f = 1$. Hence the potential effect of ART is reduced in magnitude while the direction of the effect remains the same.

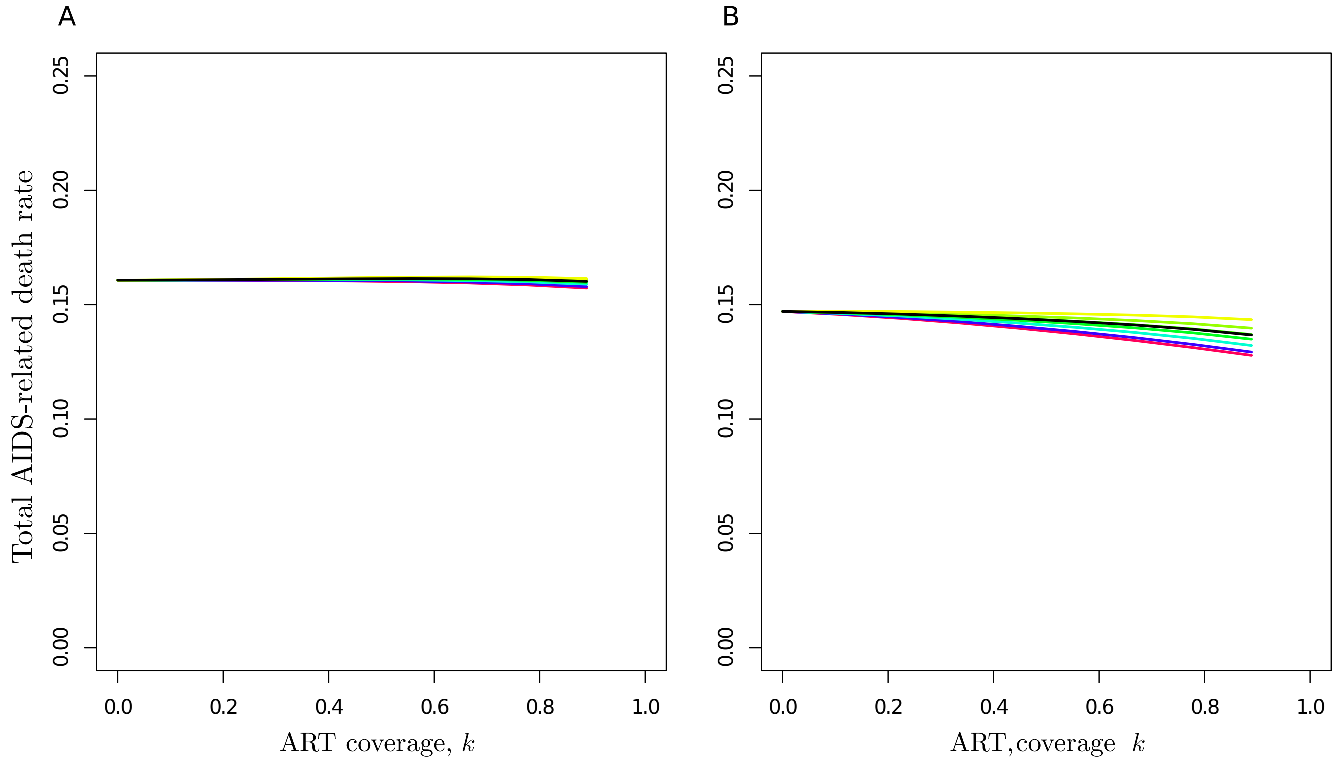

Figure S3:**The effect of ART on virulence in the short term, 50 years after the introduction of ART, when $f = 0.8$.** The results of model simulations in which ART is introduced 40 years into the epidemic are shown. Parameters and colours are as in figure 5 (main text) but with the heritability parameter $f = 0.8$. Recall $\frac{B_H}{A_H}$ is kept constant while $\frac{B_L}{A_L}$ is varied from low (red line) to high (yellow line). Initial conditions are as given in figure 3 (main text). A: $R_0^{S1} = 2.4, R_0^{S2} = 2.2$. B: $R_0^{S1} = 2.2, R_0^{S2} = 2.4$. Total number of AIDS-related deaths per untreated infected person per year is plotted on the y axis. Note that points are not plotted for $k = 1$ because when all patients are treated there are no AIDS-related deaths and so overall virulence is not well defined at this point. Since each strain now gives rise to a mixture of high and low virulence infections, changes in the strain balance have a less severe effect on overall virulence and so the potential effect of ART is reduced compared to in figure 6 (main text) when $f = 1$. The direction of the ART-effect is to decrease overall virulence at this point in time for all parameter sets, as before.

3 Model with increased transmission in acute infection

3.1 Methods

In this model we add in an 'acute' stage of infection, A, which lasts for on average $1/\alpha$ years. Individuals in the acute classes have γ-fold higher infectiousness. When these individuals transition into the standard infected classes they are at that point subdivided unto those who will or won't later receive treatment. The ODE model equations are shown below.

$$
\begin{aligned}
\frac{dS}{dt} &= B - (\beta_H c(I_\cdot^H + \gamma A_\cdot^H) + \beta_L c(I_\cdot^L + \gamma A_\cdot^L))S/N && - \mu S \\
\frac{dA_{S1}^H}{dt} &= f(\beta_H c(I_{S1}^H + \gamma A_{S1}^H) + \beta_L c(I_{S1}^L + A_{S1}^L))S/N && - (\mu + a_H + \alpha)A_{S1}^H \\
\frac{dA_{S1}^L}{dt} &= (1-f)(\beta_H c(I_{S1}^H + \gamma A_{S1}^H) + \beta_L c(I_{S1}^L + A_{S1}^L))S/N && - (\mu + a_L + \alpha)A_{S1}^L \\
\frac{dA_{S2}^H}{dt} &= (1-f)(\beta_H c(I_{S2}^H + \gamma A_{S2}^H) + \beta_L c(I_{S2}^L + A_{S2}^L))S/N && - (\mu + a_H + \alpha)A_{S2}^H \\
\frac{dA_{S2}^L}{dt} &= f(\beta_H c(I_{S2}^H + \gamma A_{S2}^H) + \beta_L c(I_{S2}^L + A_{S2}^L))S/N && - (\mu + a_L + \alpha)A_{S2}^L \\
\frac{dI_{S1,u}^H}{dt} &= (1-k)\alpha A_{S1}^H && - (\mu + a_H)I_{S1,u}^H - mI_{S1,u}^H + mI_{S2,u}^H \\
\frac{dI_{S1,t}^H}{dt} &= k\alpha A_{S1}^H && - (\mu + p_H)I_{S1,t}^H - mI_{S1,t}^H + mI_{S2,t}^H \\
\frac{dI_{S1,u}^L}{dt} &= (1-k)\alpha A_{S1}^L && - (\mu + a_L)I_{S1,u}^L - mI_{S1,u}^L + mI_{S2,u}^L \\
\frac{dI_{S1,t}^L}{dt} &= k\alpha A_{S1}^L && - (\mu + p_L)I_{S1,t}^L - mI_{S1,t}^L + mI_{S2,t}^L \\
\frac{dI_{S2,u}^H}{dt} &= (1-k)\alpha A_{S2}^H && - (\mu + a_H)I_{S2,u}^H - mI_{S2,u}^H + mI_{S1,u}^H \\
\frac{dI_{S2,t}^H}{dt} &= k\alpha A_{S2}^H && - (\mu + p_H)I_{S2,t}^H - mI_{S2,t}^H + mI_{S1,t}^H \\
\frac{dI_{S2,u}^L}{dt} &= (1-k)\alpha A_{S2}^L && - (\mu + a_L)I_{S2,u}^L - mI_{S2,u}^L + mI_{S1,u}^L \\
\frac{dI_{S2,t}^L}{dt} &= k\alpha A_{S2}^L && - (\mu + p_L)I_{S2,t}^L - mI_{S2,t}^L + mI_{S1,t}^L \\
\frac{dT}{dt} &= p_H(I_{S1,t}^H + I_{S2,t}^H) + p_L(I_{S1,t}^L + I_{S2,t}^L) && - \mu T
\end{aligned}
\tag{5}
$$

The R_0 values for this model are calculated as follows (in the absence of ART, and using the

approximation $m = 0$ as before):

$$R_0^{S1} = \frac{\gamma\beta_H}{\mu + a_H + \alpha} + \frac{\alpha}{\mu + a_H + \alpha}\frac{\beta_H}{\mu + a_H}$$
$$R_0^{S2} = \frac{\gamma\beta_L}{\mu + a_L + \alpha} + \frac{\alpha}{\mu + a_L + \alpha}\frac{\beta_L}{\mu + a_L}$$

To examine how changing the transmission profile alters the relative importance of low CD4 transmissions we switch to using the PDE form of the model. The partial differential equations for the infected populations are no different to in the original PDE model (equations 4, main text), except that λ_i changes. Let acute infection last for A years. In the simple $m = 0$ case we are now interested in when the following inequality holds:

$$\frac{\int_{T_H}^{\infty} y_{S1}^H \mathrm{d}\tau}{\int_{T_H}^{\infty} y_{S1}^H \mathrm{d}\tau + \int_{T_L}^{\infty} y_{S2}^L \mathrm{d}\tau} > \frac{\gamma \int_0^A y_{S1}^H \mathrm{d}\tau + \int_A^{\infty} y_{S1}^H \mathrm{d}\tau}{\gamma \int_0^A y_{S1}^H + y_{S2}^L \mathrm{d}\tau + \int_A^{\infty} y_{S1}^H + y_{S2}^L \mathrm{d}\tau} \tag{6}$$

where, as before (with $m = 0$)

$$\begin{aligned} y_{S1}^H &= \lambda_{S1} S e^{-\mu\tau} \times \begin{cases} 1 & \text{if } \tau \leq T_H \\ (1-k)e^{-d_H(\tau - T_H)} & \text{if } \tau > T_H \end{cases} \\ y_{S2}^L &= \lambda_{S2} S e^{-\mu\tau} \times \begin{cases} 1 & \text{if } \tau \leq T_L \\ (1-k)e^{-d_L(\tau - T_L)} & \text{if } \tau > T_L \end{cases} \end{aligned} \tag{7}$$

Note that the end of the acute period ($t = A$) is before the start of the treatment period for both high and low virulence infections ($t = T_H, T_L$). Hence

$$\int_{T_H}^{\infty} y_{S1}^H \mathrm{d}\tau = \lambda_{S1} S \frac{e^{-\mu T_H}}{\mu + d_H}$$
$$\gamma \int_0^A y_{S1}^H \mathrm{d}\tau + \int_A^{\infty} y_{S1}^H \mathrm{d}\tau = \lambda_{S1} S \left[\frac{e^{-\mu T_H}}{\mu + d_H} + \gamma\left(\frac{1}{\mu} - \frac{1}{\mu}e^{-\mu A}\right) + \frac{1}{\mu}e^{-\mu A} - \frac{1}{\mu}e^{-\mu T_H}\right]$$

Writing (6) in terms of the parameters of the model:

$$\begin{aligned} &\left(\lambda_{S1} S \frac{e^{-\mu T_H}}{\mu + d_H}\right)\left(\lambda_{S1} S \left[\frac{e^{-\mu T_H}}{\mu + d_H} + \gamma\left(\frac{1}{\mu} - \frac{1}{\mu}e^{-\mu A}\right) + \frac{1}{\mu}e^{-\mu A} - \frac{1}{\mu}e^{-\mu T_H}\right]\right. \\ &\qquad\qquad \left. + \lambda_{S2} S \left[\frac{e^{-\mu T_L}}{\mu + d_L} + \gamma\left(\frac{1}{\mu} - \frac{1}{\mu}e^{-\mu A}\right) + \frac{1}{\mu}e^{-\mu A} - \frac{1}{\mu}e^{-\mu T_L}\right]\right) > \\ &\left(\lambda_{S1} S \frac{e^{-\mu T_H}}{\mu + d_H} + \lambda_{S2} S \frac{e^{-\mu T_L}}{\mu + d_L}\right)\left(\lambda_{S1} S \left[\frac{e^{-\mu T_H}}{\mu + d_H} + \gamma\left(\frac{1}{\mu} - \frac{1}{\mu}e^{-\mu A}\right) + \frac{1}{\mu}e^{-\mu A} - \frac{1}{\mu}e^{-\mu T_H}\right]\right) \end{aligned}$$

After multiplying out, the $\lambda_{S1}\lambda_{S1}$ terms all cancel and we can then divide by $\lambda_{S1}\lambda_{S2}S^2$ to get:

$$
\begin{aligned}
&\left(\frac{e^{-\mu T_H}}{\mu + d_H}\right)\left(\left[\gamma\left(\frac{1}{\mu} - \frac{1}{\mu}e^{-\mu A}\right) + \frac{1}{\mu}e^{-\mu A} - \frac{1}{\mu}e^{-\mu T_L}\right]\right) > \\
&\qquad\qquad \left(\frac{e^{-\mu T_L}}{\mu + d_L}\right)\left(\left[\gamma\left(\frac{1}{\mu} - \frac{1}{\mu}e^{-\mu A}\right) + \frac{1}{\mu}e^{-\mu A} - \frac{1}{\mu}e^{-\mu T_H}\right]\right) \\
\Rightarrow \quad & \frac{\mu + d_L}{\mu + d_H} > \frac{\left(\gamma - \gamma e^{-\mu A} + e^{-\mu A}\right) e^{\mu T_H} - 1}{\left(\gamma - \gamma e^{-\mu A} + e^{-\mu A}\right) e^{\mu T_L} - 1}
\end{aligned}
$$

3.2 Results: Magnitude and direction of the ART-effect

To model a scenario in which in the absence of ART approximately 50% of all transmissions occur in acute infection, we take $\gamma = 13$ and $\alpha = 2$ (or equivalently, $A = 1/2$, so the increased transmission of acute infection lasts for 6 months). Supplementary figures 4–6 show the results of this 'increased acute phase infectivity' model using these values for γ and α. Note that as we increase the proportion of transmissions that are occurring in acute infection, the importance of low CD4 transmissions to the force of infection of both strains decreases. Hence when 50% of transmissions occur in acute infection ART has a lesser effect on viral dynamics and higher coverage is needed to see effects of the same magnitude as before (see supplementary figures 5 and 6 and compare with figures 5 and 6 in the main text).

With these parameters, the condition for ART to benefit the low virulence strain is:

$$
\frac{\mu + d_L}{\mu + d_H} > \frac{1.15 e^{\mu T_H} - 1}{1.15 e^{\mu T_L} - 1}
$$

(where as when we previously assumed constant probability of transmission throughout infection the RHS was $(e^{\mu T_H} - 1)/(e^{\mu T_L} - 1)$). Since $e^{\mu T_H}$ and $e^{\mu T_L}$ are both a little greater than 1, the fraction on the RHS is larger than previously. Hence, when we account for increased transmission in acute infection, then d_L and d_H have to be closer in size than they did before in order to see a benefit to the low virulence strain. Or, in other words, there are fewer cases in which the effect of ART is to drive a decrease in virulence (as seen in supplementary figure 5 compared with figure 5 (main text)).

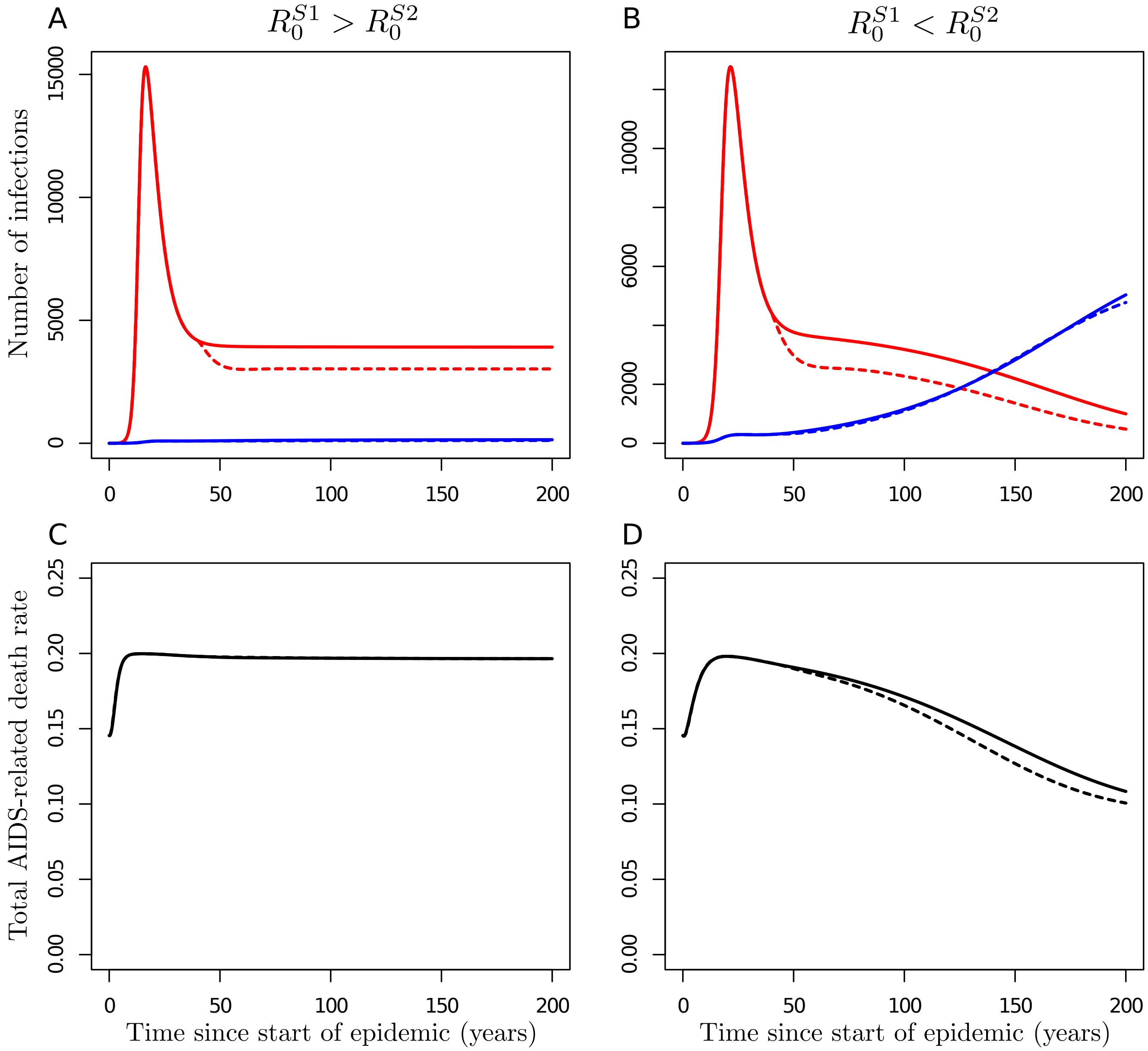

Figure S4:**Behaviour of the model with 50% of transmissions occurring in the first 6 months of infection, with and without ART.** Parameters and initial conditions are as in figure 3 (main text) and with $\alpha = 2, \gamma = 13$. A, C: In the absence of ART $R_0^{S1} = 2.0, R_0^{S2} = 1.8$. B, D: In the absence of ART $R_0^{S1} = 1.8, R_0^{S2} = 2.0$. Solid lines show the output of the model without ART while dotted lines plot trajectories for the model when ART is introduced at 30% coverage 40 years into the epidemic. A, B: The number of strain S1 infections through time is shown in red and the number of strain S2 infections in blue. With increased transmission earlier in infection the peak of the epidemic occurs earlier, even given lower R_0 values used in this figure compared to figure 3 in the main text. C, D: In this model equilibrium is reached earlier and so the decrease of virulence in (D) occurs faster. The effect of ART on virulence is of smaller magnitude compared to the original model with constant transmission profile.

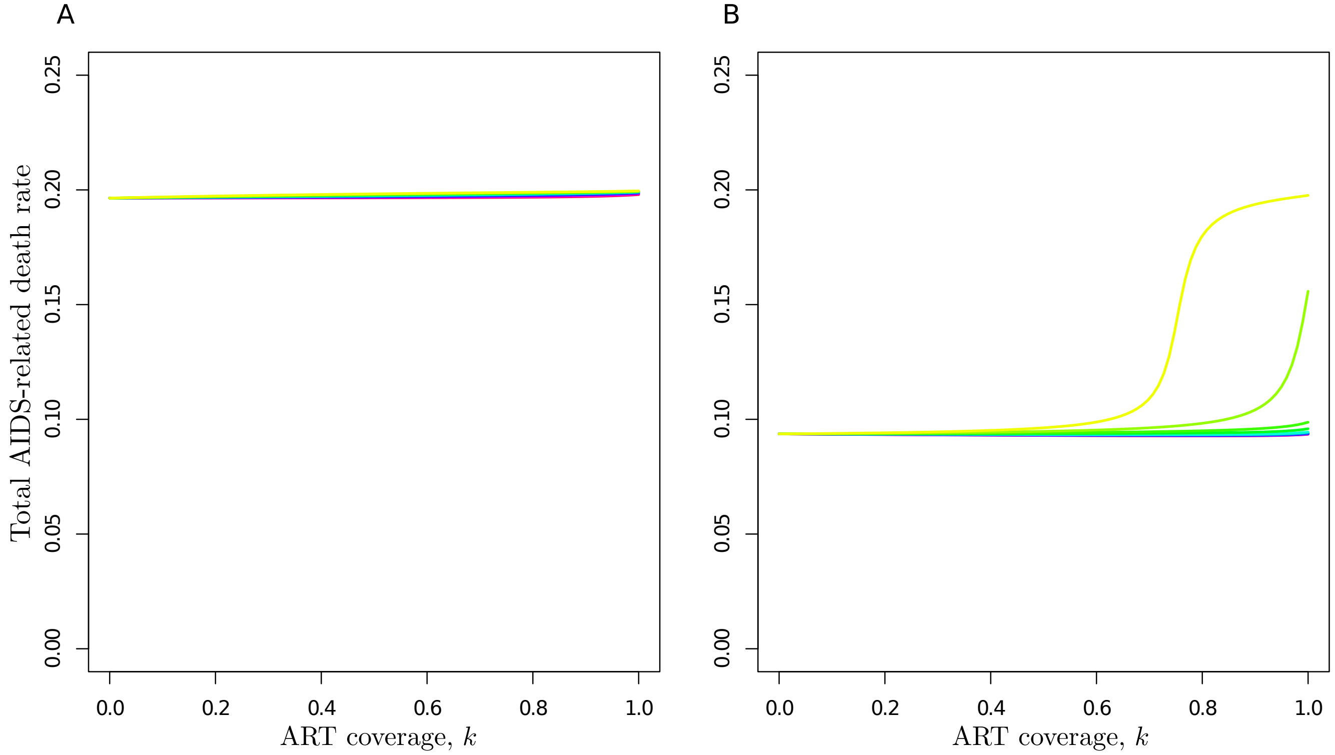

Figure S5: **The relationship between ART coverage and virulence at equilibrium, in the model with 50% of transmissions occurring in the first 6 months of infection.** Parameters and colours as in figure 5 (main text) plus $\alpha = 2, \gamma = 13$. Recall $\frac{B_H}{A_H}$ is kept constant while $\frac{B_L}{A_L}$ is varied from low (red line) to high (yellow line). A: $R_0^{S1} = 2.0, R_0^{S2} = 1.8$. B: $R_0^{S1} = 1.8, R_0^{S2} = 2.0$. Total number of AIDS-related deaths per untreated infected person per year is plotted on the y axis. Since transmissions late in infection are now of lesser relative importance to both strains, ART very rarely has a large effect on virulence. There are fewer cases in which the effect of ART is to drive a decrease in virulence.

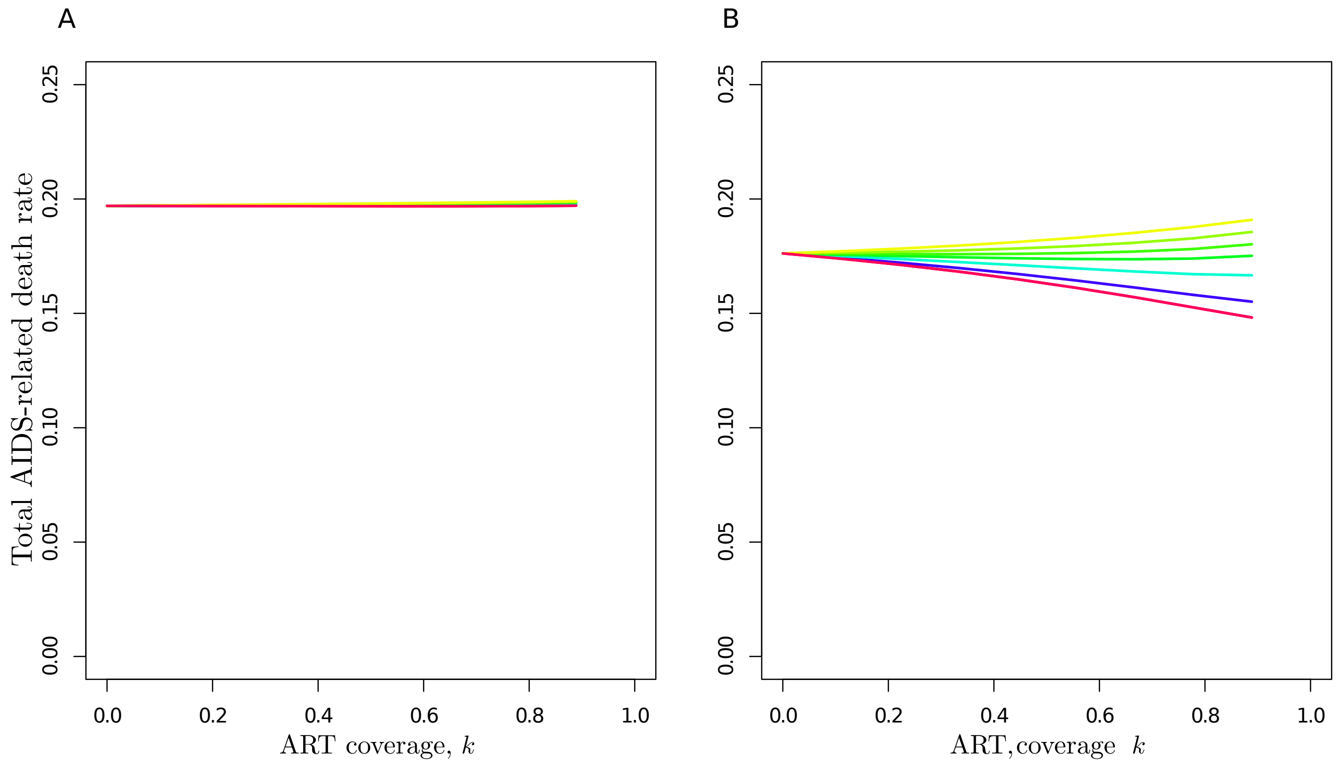

Figure S6: **The effect of ART on virulence in the short term, 50 years after the introduction of ART, in the model with 50% of transmissions occurring in the first 6 months of infection.** The results of model simulations in which ART is introduced 40 years into the epidemic are shown. Parameters and colours are as in figure 5 (main text) plus $\alpha = 2, \gamma = 13$. Recall $\frac{B_H}{A_H}$ is kept constant while $\frac{B_L}{A_L}$ is varied from low (red line) to high (yellow line). Initial conditions are as given in figure 3 (main text). A: $R_0^{S1} = 2.0, R_0^{S2} = 1.8$. B: $R_0^{S1} = 1.8, R_0^{S2} = 2.0$. Total number of AIDS-related deaths per untreated infected person per year is plotted on the y axis. As seen in the equilibrium calculations, the effect of ART on virulence in this model is smaller in magnitude compared to the model with constant transmission profile (figure 6, main text) and there are fewer cases in which the effect of ART is to drive a decrease in virulence.

4 Modelling increased transmission amongst a subsection of the population

4.1 Methods

For this extension of the model, we split the population into a 'core' and 'periphery', where there will be much higher contact between individuals in the core and the core will be much smaller than the periphery. We assume no movement between the core and periphery compartments. So the basic structure of the model is:

$$\begin{aligned} \text{Core}: \quad & S_c \xrightarrow{\lambda_c} I_c \\ \text{Periphery}: \quad & S_p \xrightarrow{\lambda_p} I_p \end{aligned}$$

where, now specifying the viral strain, i,

$$\begin{aligned} \lambda_{i,c} &= \beta_H c_c \frac{I^H_{i,c}}{S_c + I_c} + \beta_L c_c \frac{I^L_{i,c}}{S_c + I_c} + \beta_H c_b \frac{I^H_{i,p}}{S_p + I_p} + \beta_L c_b \frac{I^L_{i,p}}{S_p + I_p} \\ \lambda_{i,p} &= \beta_H c_p \frac{I^H_{i,p}}{S_p + I_p} + \beta_L c_p \frac{I^L_{i,p}}{S_p + I_p} + \beta_H c_b \frac{I^H_{i,c}}{S_c + I_c} + \beta_L c_b \frac{I^L_{i,c}}{S_c + I_c} \end{aligned}$$

and we now have three contact rates: c_c is the contact rate within the core, c_p is the contact rate within the periphery, and c_b is the contact rate *between* the core and the periphery. Hence $c_c > c_p, c_b$. The spread of the infection within each compartment is described by the equations in (1), with λ_i replaced by $\lambda_{i,c}$ or $\lambda_{i,p}$ as appropriate. We introduce ART to the core only.

Let $c_b = c_p$, so it is only within the core that there are higher contact rates. We wish to use this model to simulate the dynamics of generalised and concentrated epidemics and compare the two. For the generalised epidemic we let $c_p = \frac{1}{2}c_c$ and then for the concentrated epidemic $c_p = \frac{1}{100}c_c$.

4.2 Comparison of generalised and concentrated epidemics

The results in figure 7 show that in the generalised epidemic the dynamics of the core and the periphery are almost identical. This situation is not much different from our original model. Therefore it is not surprising that virulence change over time is very similar to in figure 3 (main text), both in the absence and presence of ART.

In contrast, in figure 8 we see that in the concentrated epidemic the dynamics of the periphery lag behind those of the core and the fraction of individuals infected in the periphery is more than 10 times smaller than in the core. Note that in the concentrated model the forces of infection are dominated by the following terms:

$$\begin{aligned} \lambda_{i,c} &\approx \beta_H c_c \frac{I^H_{i,c}}{S_c + I_c} + \beta_L c_c \frac{I^L_{i,c}}{S_c + I_c} \\ \lambda_{i,p} &\approx \beta_H c_b \frac{I^H_{i,c}}{S_c + I_c} + \beta_L c_b \frac{I^L_{i,c}}{S_c + I_c} \end{aligned}$$

Hence the spread of infection in the core is influenced very little by the periphery, whereas in the periphery the incidence of infection is almost entirely determined by the prevalence of infection in the core and the force of infection is low. These observations explain the strain dynamics seen in figure 8.

Despite the differences in dynamics between the core and periphery, we see in figure 8 that patterns of virulence change in the first 200 years of the epidemic are very similar to in the generalised epidemic, both in the absence and presence of ART. We wish to gain an understanding of what we would expect the ART-effect in the long term (at equilibrium) to be in the concentrated model. First note that in general we would expect the magnitude of the effect to be slightly greater because we are targeting therapy at the individuals who contribute most to onward transmission (the core). We would expect the direction of the ART effect to be similar to that in the original model (since the relative importance of 'low CD4' transmissions to each strain hasn't changed). so long as the ratio of high:low SPVL infections in the core (where ART is applied) is similar to that in the periphery. We can use the approximated forces of infection above to do a straightforward calculation of the equilibrium position of the model (in the absence of ART):

$$\frac{\mathrm{d}I^H_{S1,c}}{dt} = 0 \quad \Rightarrow \quad \beta_H c_c \frac{I^H_{S1,c}}{N_c} S_c + \beta_L c_c \frac{I^L_{S1,c}}{N_c} S_c - (\mu + a_H + m) I^H_{S1,c} + m I^H_{S2,c} = 0$$

$$\Rightarrow \quad \frac{\beta_H c_c I^H_{S1,c} + \beta_L c_c I^L_{S1,c}}{(\mu + a_H + m) I^H_{S1,c} - m I^H_{S2,c}} = \frac{N_c}{S_c}$$

$$\frac{\mathrm{d}I^L_{S1,c}}{dt} = 0 \quad \Rightarrow \quad -(\mu + a_H + m) I^L_{S1,c} + m I^L_{S2,c} = 0$$

$$\Rightarrow \quad \frac{m}{\mu + a_H + m} I^H_{S1,c} = I^H_{S2,c}$$

$$\Rightarrow \quad \frac{\beta_H c_c I^H_{S1,c} + \beta_L c_c I^L_{S1,c}}{(\mu + a_H + m) I^H_{S1,c} - \frac{m^2}{\mu + a_H + m} I^H_{S1,c}} = \frac{N_c}{S_c}$$

Similar calculations for S2 in the core and S1 and S2 in the periphery yield

$$\frac{\beta_H c_c I^H_{S2,c} + \beta_L c_c I^L_{S2,c}}{(\mu + a_L + m) I^L_{S2,c} - \frac{m^2}{\mu + a_L + m} I^L_{S2,c}} = \frac{N_c}{S_c}$$

$$\frac{\beta_H c_b I^H_{S1,c} + \beta_L c_b I^L_{S1,c}}{(\mu + a_H + m) I^H_{S1,p} - \frac{m^2}{\mu + a_H + m} I^H_{S1,p}} = \frac{N_c}{S_p}$$

$$\frac{\beta_H c_b I^H_{S2,c} + \beta_L c_b I^L_{S2,c}}{(\mu + a_L + m) I^L_{S2,p} - \frac{m^2}{\mu + a_L + m} I^L_{S2,p}} = \frac{N_c}{S_p}.$$

Combining the core and periphery pairs of equations and rearranging gives

$$\frac{(\mu + a_H + m) I^H_{S1,c} - \frac{m^2}{\mu + a_H + m} I^H_{S1,c}}{(\mu + a_L + m) I^L_{S2,c} - \frac{m^2}{\mu + a_L + m} I^L_{S2,c}} = \frac{(\mu + a_H + m) I^H_{S1,p} - \frac{m^2}{\mu + a_H + m} I^H_{S1,p}}{(\mu + a_L + m) I^L_{S2,p} - \frac{m^2}{\mu + a_L + m} I^L_{S2,p}}.$$

When $m << 1$, as in the original model, this approximates to $I^H_c / I^H_p = I^L_c / I^L_p$. In other words the ratio of high:low SPVL infections is approximately the same in the core as in the population

as a whole. Hence we would expect the direction of the ART-effect on virulence at equilibrium to be similar to that in the original model.

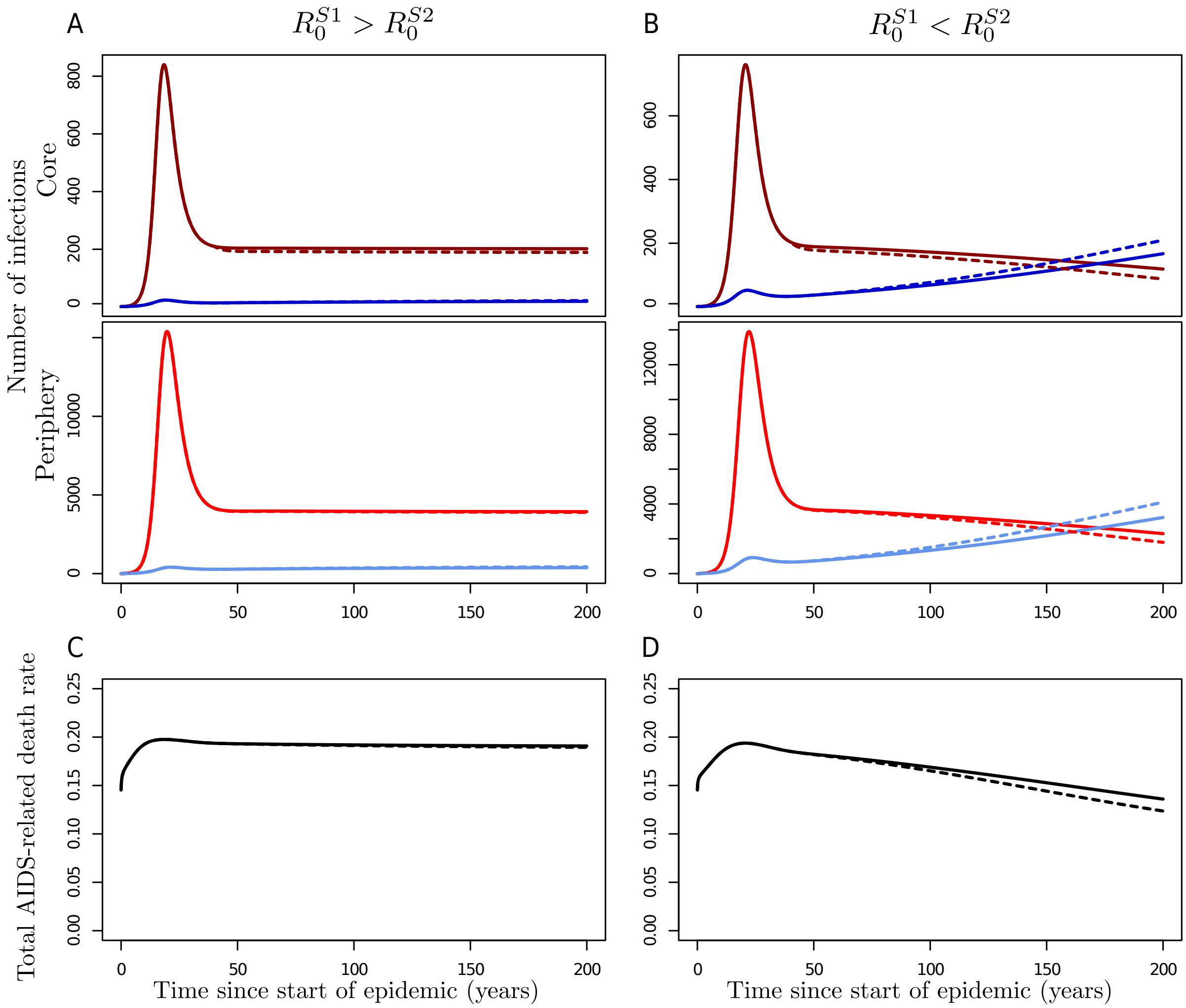

Figure S7: **Behaviour of the core-periphery model under the assumptions of a generalised epidemic, with and without ART.** Parameters are as in table 1 but with the differing contact rates $c_c = 1, c_p = c_b = 0.5$. The core is 20 times smaller than the periphery, so $S_c(0) = 2000, S_p(0) = 40000$. Initially there are no infections in the periphery, but in the core $I^H_{S1,u}(0) = I^L_{S2,u}(0) = 1$ and all other infected populations are 0 initially. A, C: In the absence of ART, the R_0s of the core (where $c = 1$) are set at $R_0^{S1} = 2.4, R_0^{S2} = 2.2$. In other words, we choose β_H, β_L such that $2.4 = \beta_H/(\mu + a_H)$, $2.2 = \beta_L/(\mu + a_L)$. B, D: In the absence of ART, the R_0s of the core ($c = 1$) are set at $R_0^{S1} = 2.2, R_0^{S2} = 2.4$. Solid lines show the output of the model without ART while dotted lines plot trajectories for the model when ART is introduced 40 years into the epidemic. Only the core is treated (at coverage of 30%). A, B: The number of strain S1 infections through time is shown in red and the number of strain S2 infections in blue. In this simulation of a generalised epidemic, though the dynamics are driven by transmission in the core, the dynamics in the periphery closely match those in the core. C, D: Patterns of virulence change over time are qualitatively similar to the original model, though the peak is smaller and the magnitude of the ART-effect is lesser since only the core is treated.

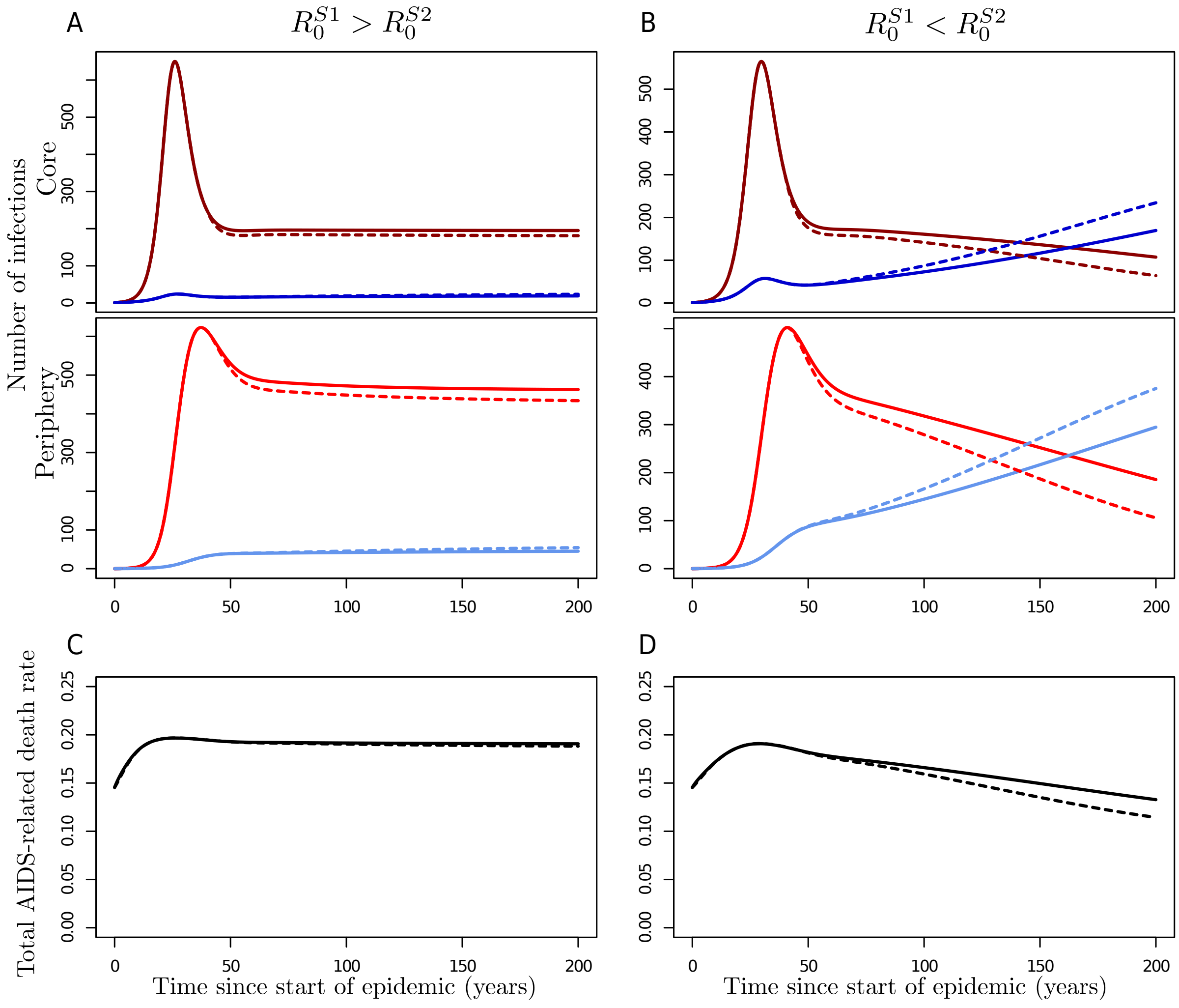

Figure S8: **Behaviour of the core-periphery model under the assumptions of a concentrated epidemic, with and without ART.** Parameters are as in table 1 but with the differing contact rates $c_c = 1, c_p = c_b = 0.01$. The core is 20 times smaller than the periphery, so $S_c(0) = 2000, S_p(0) = 40000$ and also $B_c = 50$ and $B_p = 1000$. Initially there are no infections in the periphery, but in the core $I^H_{S1,u}(0) = I^L_{S2,u}(0) = 1$ and all other infected populations are 0 initially. A, C: In the absence of ART, the R_0s of the core ($c = 1$) are set at $R_0^{S1} = 2.4, R_0^{S2} = 2.2$. B, D: In the absence of ART, the R_0s of the core ($c = 1$) are set at $R_0^{S1} = 2.2, R_0^{S2} = 2.4$. Solid lines show the output of the model without ART while dotted lines plot trajectories for the model when ART is introduced 40 years into the epidemic. Only the core is treated (at coverage of 30%). A, B: The number of strain S1 infections through time is shown in red and the number of strain S2 infections in blue. Compared to the generalised epidemic the peak of infections is later due to reduced transmission in the periphery, and the number of infections is much lower in the periphery. The force of infection in the periphery is very dependent on the proportion of infected individuals in the core and the shape of the epidemic peak reflects this. C, D: Patterns of virulence change are still very similar to those seen in the original model, except that the low virulence strain (S2) starts to dominate earlier in (D) than in fig 3D (main text).

5 Modelling an ART treatment protocol where all patients are treated as soon as possible

5.1 Methods

To model a set of treatment guidelines where all individuals are treated as soon as possible (i.e. as soon as they are identified as HIV-positive) we use the original ODE model with ART, as given in equations 2, main text, and set $p_H = p_L$. In other words, identification of infection and resulting treatment is independent of disease course. In the figures that follow (figures 9 and 10) we vary the average time from infection to identification of infection between 6 months and 4 years and look at the resulting ART-effect at equilibrium (figure 9) and in the short-term (figure 10).

5.2 Results

Under this treatment protocol, the low virulence strain is always affected most when ART is introduced because a greater proportion of its onward transmissions are effectively removed (compared to the onward transmissions of the high virulence strain). Therefore in the long term (at equilibrium), ART always drives an increase in virulence, whatever the value of the parameter p_j. The magnitude of the effect is smaller when p_j is large, i.e. when patients are treated particularly early in infection, because in that case both strains are (more equally) heavily affected by ART.

In the short term, the effect of ART is either to benefit the high virulence strain (S1), in which case the magnitude is small because S1 dominates in the absence of ART anyway, or to benefit the low virulence strain. However, the benefit to the low virulence strain occurs in the (unrealistic) cases where ART is causing the eventual extinction of both strains – since the turnover of the S2-infected population is lower it decays at a slower rate and so the average virulence of an infection rises in these cases.

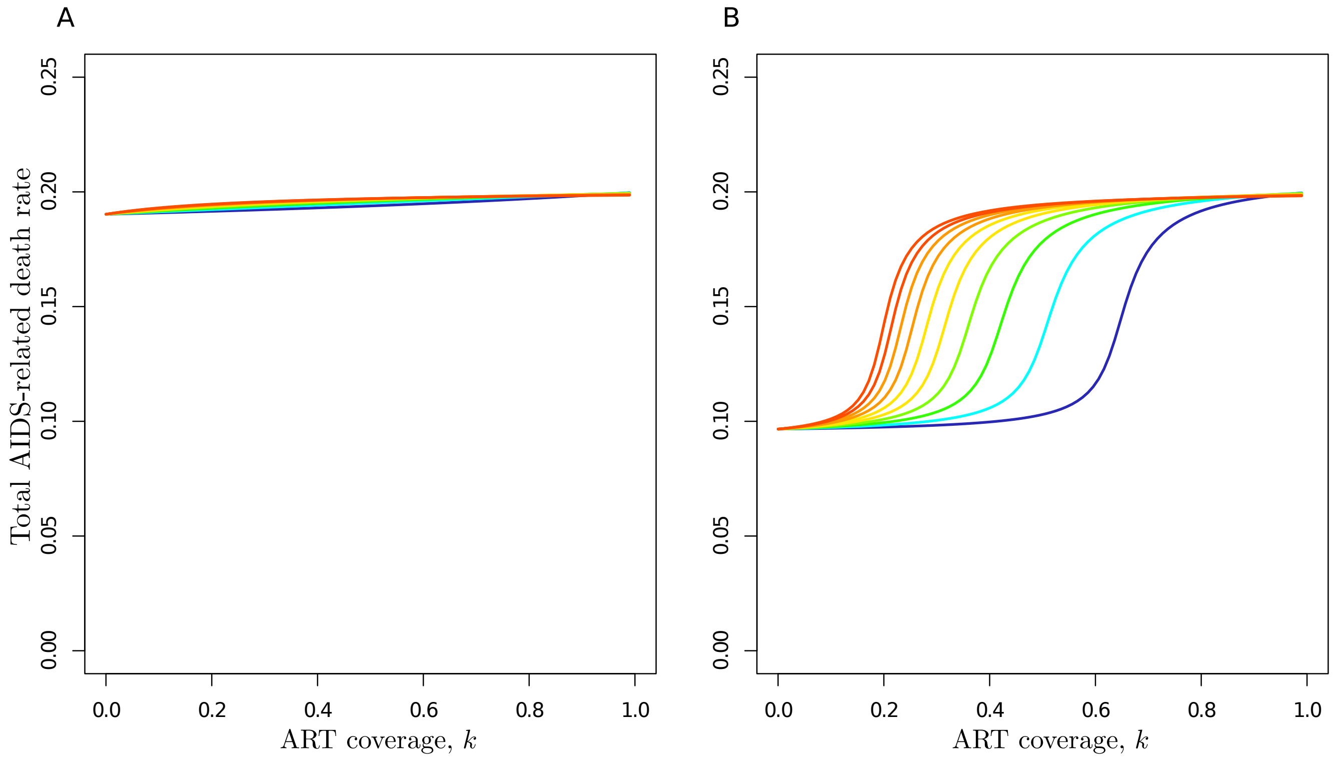

Figure S9: **The relationship between ART coverage and virulence at equilibrium when patients are treated as soon as possible after infection, regardless of disease progression**. A: $R_0^{S1} = 2.4, R_0^{S2} = 2.2$. B: $R_0^{S1} = 2.2, R_0^{S2} = 2.4$. Total number of AIDS-related deaths per untreated infected person per year is plotted on the y axis. Treating independent of disease progression translates to setting $p_L = p_H$ in the ODE model. Different coloured lines here represent different rates at which individuals in the 'treated' class are treated, from $p_j = 2$ i.e. an average of 6 months of infection before initiating ART (blue) to $p_j = 1/4$ i.e. an average of 4 years of infection before initiating ART (red). The remaining parameters are the same as in Table 1 (main text). Note that when everyone is treated almost immediately, both strains take a big hit to onward transmission, where as when individuals are treated after four years the low virulence strain, S2, takes a much bigger hit, hence overall virulence is higher the lower p_j is.

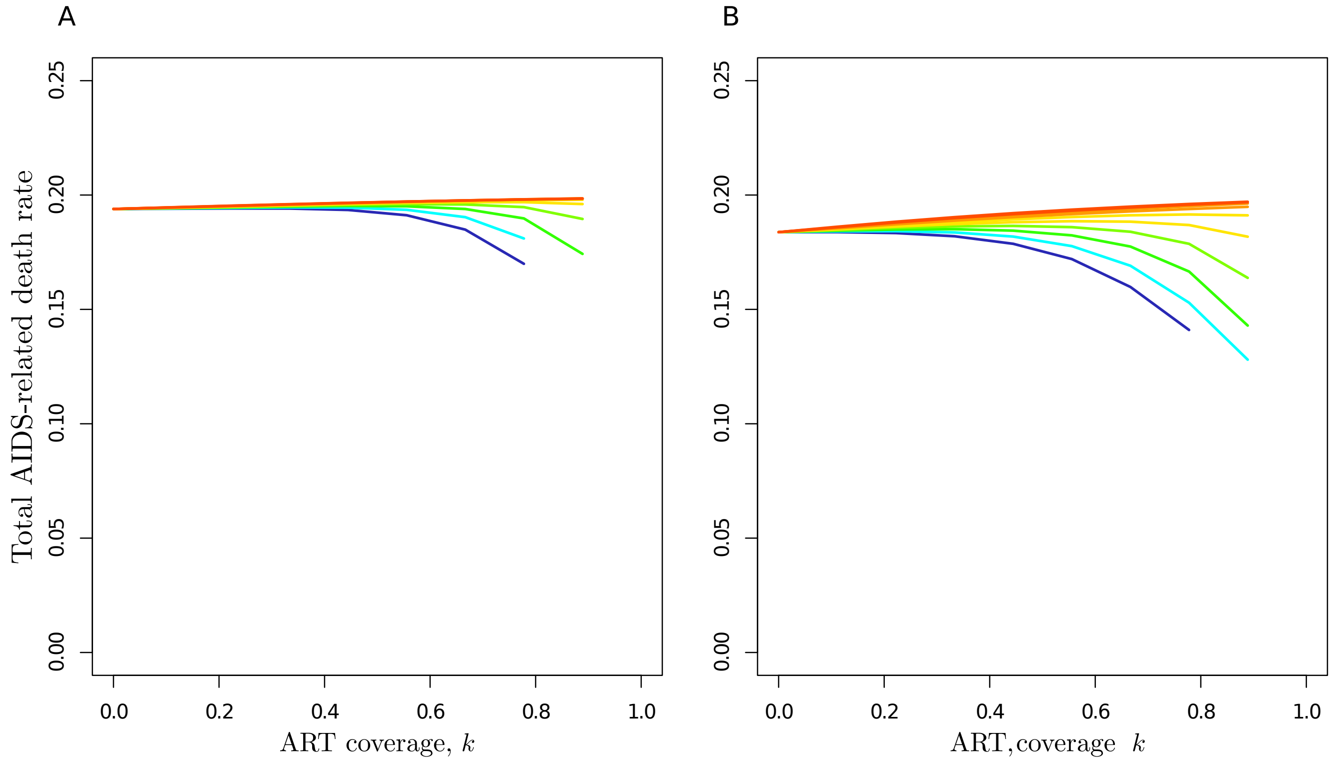

Figure S10: **The short term effect of ART on virulence, in the model where patients are treated as soon as possible after infection, regardless of disease progression**. The results of model simulations at 90 years (where ART is introduced 40 years into the epidemic) are shown, with total number of AIDS-related deaths per untreated infected person per year once again used as a proxy for virulence A: $R_0^{S1} = 2.4, R_0^{S2} = 2.2$. B: $R_0^{S1} = 2.2, R_0^{S2} = 2.4$. As in supplementary figure 9, different coloured lines represent different rates at which individuals in the 'treated' class are treated, from $p_j = 2$ i.e. an average of 6 months of infection before initiating ART (blue) to $p_j = 1/4$ i.e. an average of 4 years of infection before initiating ART (red). The remaining parameters and initial conditions are the same as in figure 3 and table 1 (main text). When p_j is low then even at this stage in the epidemic the effect of ART is to benefit the high virulence strain (S1). For higher values of p_j and ART coverage, k, ART results in the eventual extinction of both strains. Since the death rate of the S2-infected population is lower than that of the S1-infected population this can lead to a rise in the proportion of S2 compared to S1 -infected individuals in the period leading to extinction, and hence a decrease in virulence by this measure. The points representing parameter sets where this extinction has already occurred at 90 years $(I_{S1}(90) + I_{S2}(90) < 1)$ are not plotted.

6 Additional sensitivity analyses

In this section we conduct sensitivity analyses in which we vary the R_0 values (figures 11–13), the timing of ART initiation relative to the peak of the epidemic (figures 14 & 15) and the initial conditions (figure 16). These sensitivity analyses are based on the original model as presented in the main text. Table 1 summarises the results of all these sensitivity analyses and the analyses in sections 2–4 in terms of the effect of the various modifications on the key results regarding virulence evolution and the ART-effect.

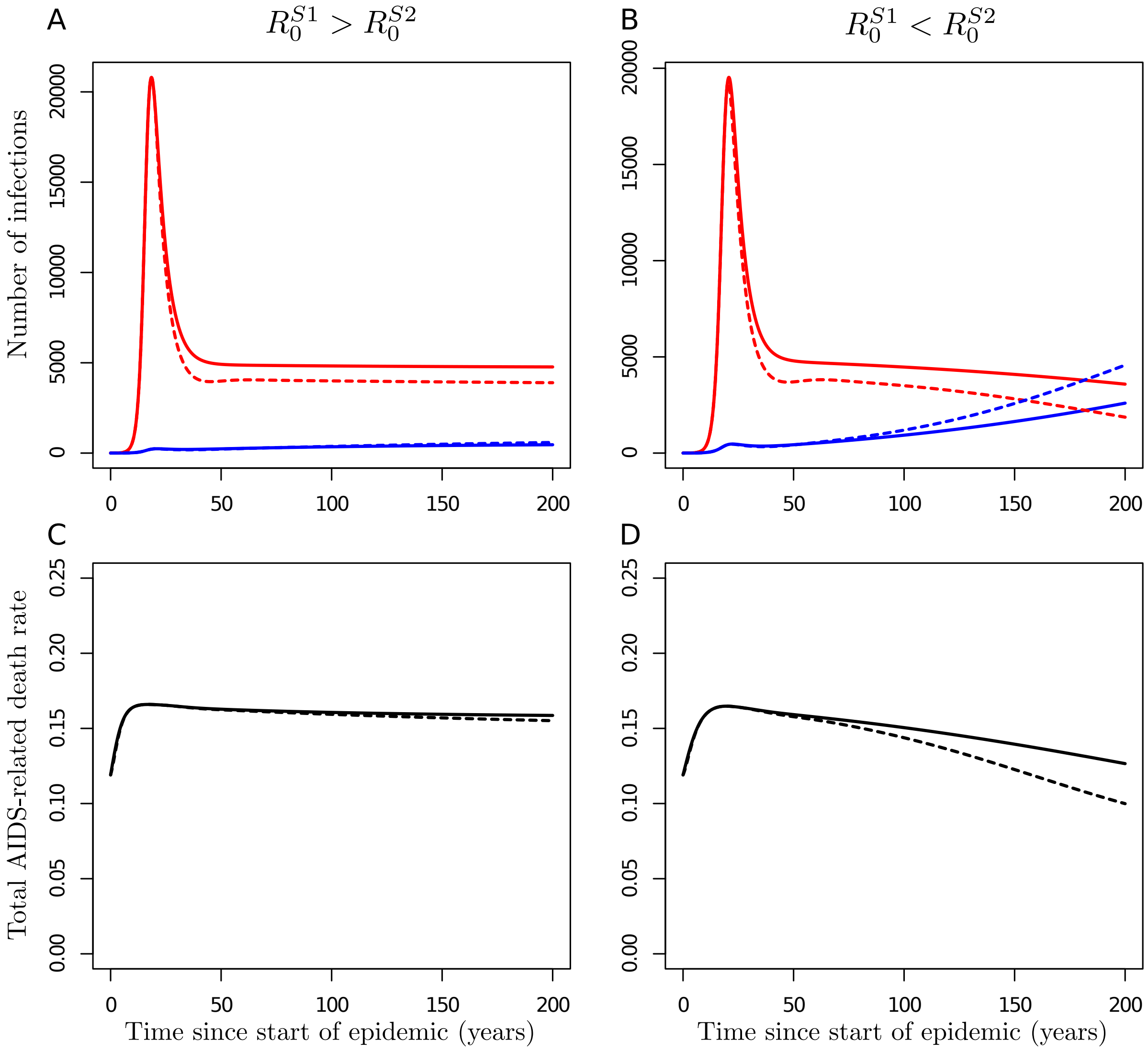

Figure S11: **Behaviour of the model where both strains have particularly high R_0s, with and without ART.** Parameters and initial conditions are as in table 1 and figure 3 (main text), except for mortality and progression rates of infected individuals which are in general lower in this simulation ($a_H = 1/6, a_L = 1/14, p_H = 1/4, p_L = 1/12$). A, C: In the absence of ART, the R_0s are set at $R_0^{S1} = 4.4, R_0^{S2} = 4.0$. B, D: In the absence of ART, the R_0s are set at $R_0^{S1} = 4.0, R_0^{S2} = 4.4$. Solid lines show the output of the model without ART while dotted lines plot trajectories for the model when ART is introduced at 30% coverage 25 years into the epidemic. A, B: The number of strain S1 infections through time is shown in red and the number of strain S2 infections in blue. The peak of the epidemic now occurs between 20 and 25 years after the start and is slightly more severe than in the original model. Otherwise the dynamics are similar. C, D: Patterns of change in virulence are qualitatively similar to the original model and occur on the same order-of-magnitude timescales.

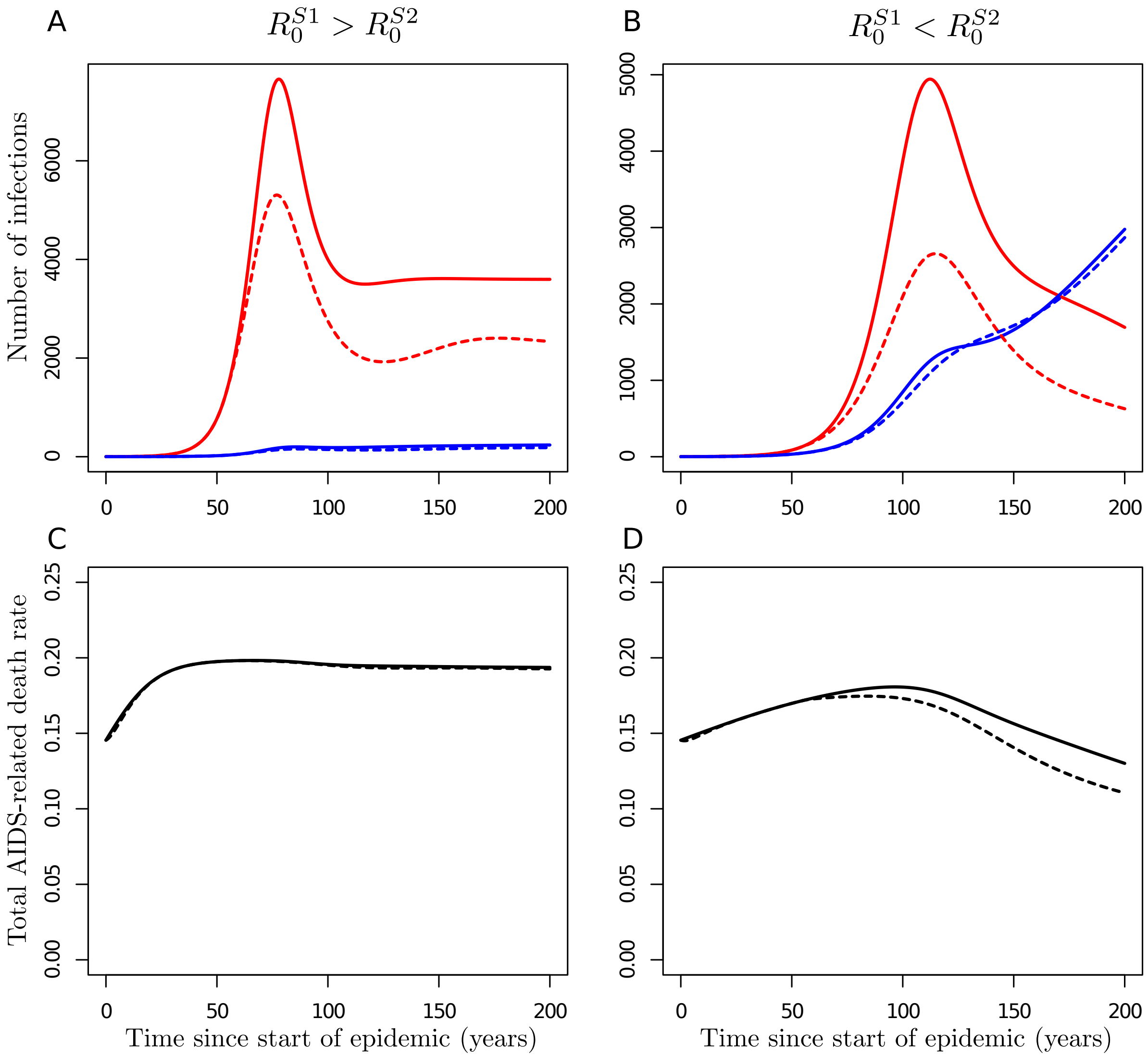

Figure S12: **Behaviour of the model where both strains have particularly low R_0s, with and without ART.** Parameters and initial conditions are as in table 1 and figure 3 (main text). A, C: In the absence of ART, the R_0s are set at $R_0^{S1} = 1.6, R_0^{S2} = 1.4$. B, D: In the absence of ART, the R_0s are set at $R_0^{S1} = 1.4, R_0^{S2} = 1.6$. Solid lines show the output of the model without ART while dotted lines plot trajectories for the model when ART is introduced at 30% coverage 55 years into the epidemic. The number of strain S1 infections through time is shown in red and the number of strain S2 infections in blue. A, B: The peak of the epidemic now occurs much later and the progress of the epidemic is in general much slower. In the case where $R_0^{S1} < R_0^{S2}$, the high virulence strain is unable to spread through the population as quickly (B) and its domination in the early stages is significantly reduced compared to in simulations with higher R_0s. C, D: Despite this, patterns of virulence change are still quantitatively similar - a first-phase rise in virulence is followed by a second phase decline (though this is modest in (C)), and the third phase is determined by the balance of the R_0s.

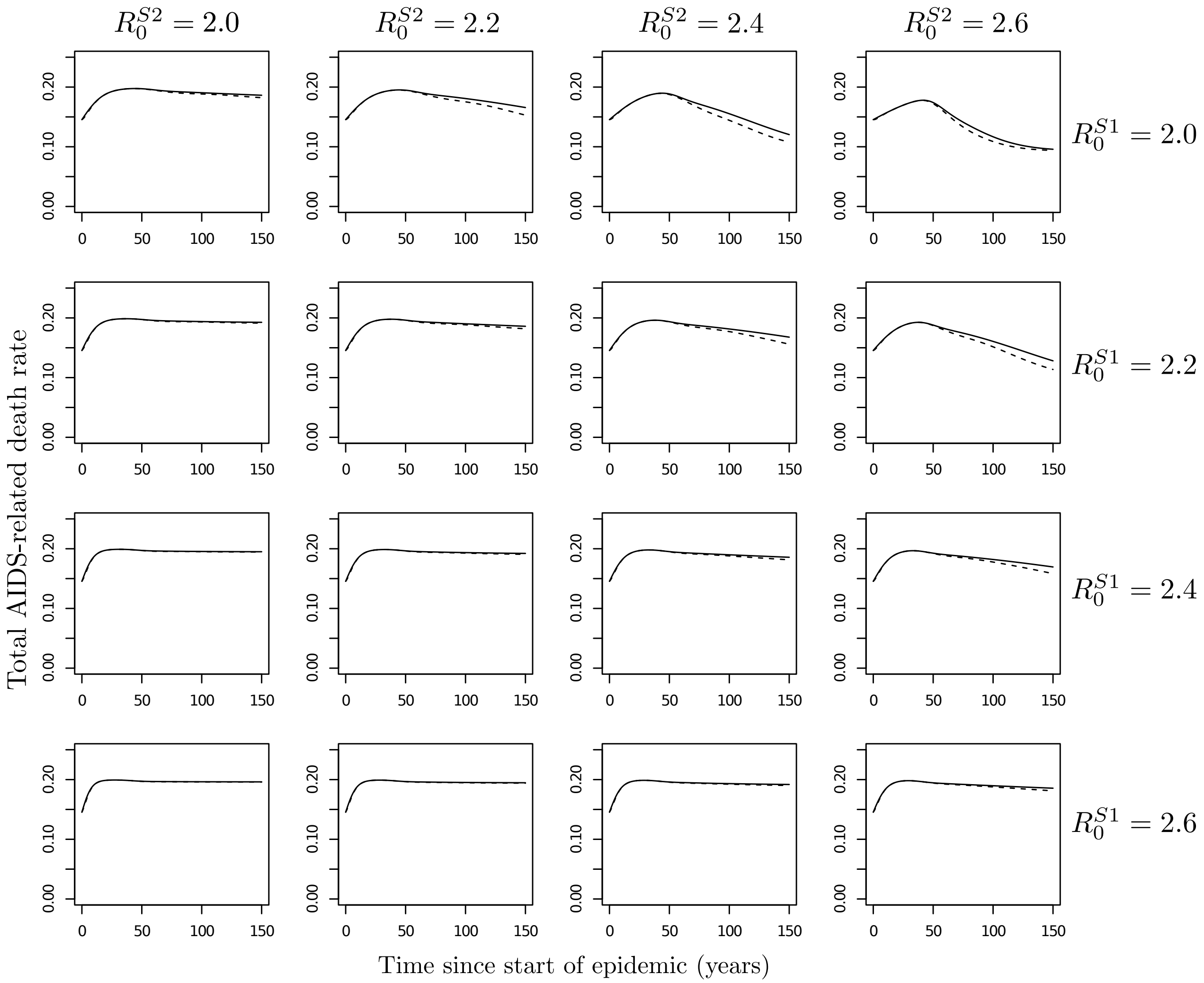

Figure S13: **Patterns of virulence change in the first 150 years of the epidemic for a range of combinations of R_0 values for the two strains**. Total number of AIDS-related deaths per untreated infected person per year is plotted on the y axis. Parameters and initial conditions are as given in table 1 and figure 3 (main text), except for the R_0 values which are varied from 2 to 2.6. Changes in virulence are quantitatively different for different R_0 combinations but the patterns of change through time are consistent. There is a decrease in virulence in the second half-century of the epidemic in all cases, this is most severe when R_0^{S2} is much greater than R_0^{S1} and very slight when R_0^{S1} is greater than R_0^{S2}. ART always results in a decrease in virulence (compared to the model with no ART) and this ART effect is largest when R_0^{S2} is a little larger than R_0^{S1}.

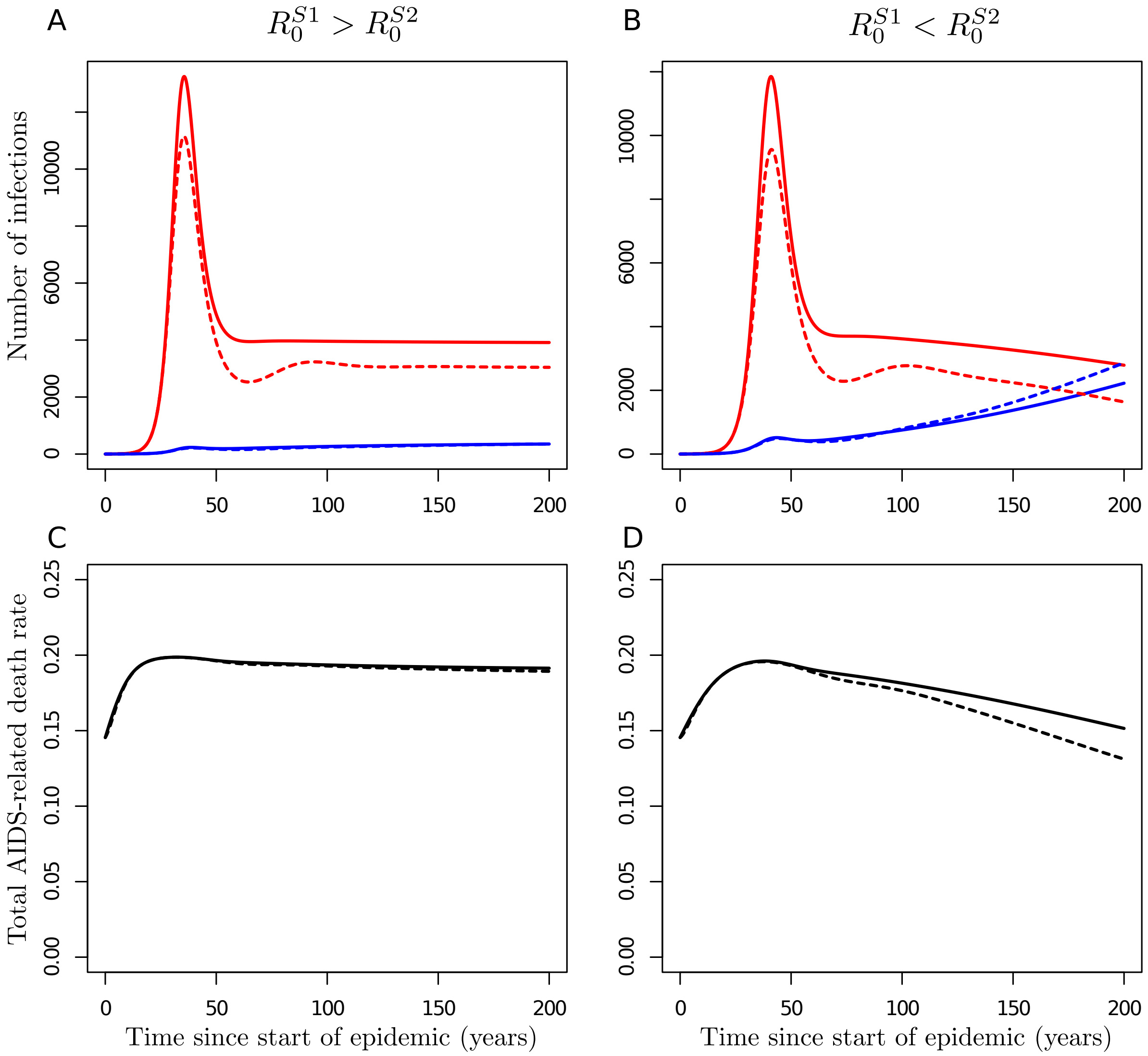

Figure S14:**Behaviour of the model without ART compared to the model where ART is introduced before the epidemic peak.** Parameters and initial conditions are as in table 1 and figure 3 (main text), including R_0 values. Solid lines show the output of the model without ART while dotted lines plot trajectories for the model when ART is introduced at 30% coverage 25 years into the epidemic. The number of strain S1 infections through time is shown in red and the number of strain S2 infections in blue. Despite ART being introduced 15 years earlier, patterns of virulence change in this simulation, measured as total AIDS-related deaths per untreated infected person per year, are almost identical to those in figure 3 (main text).

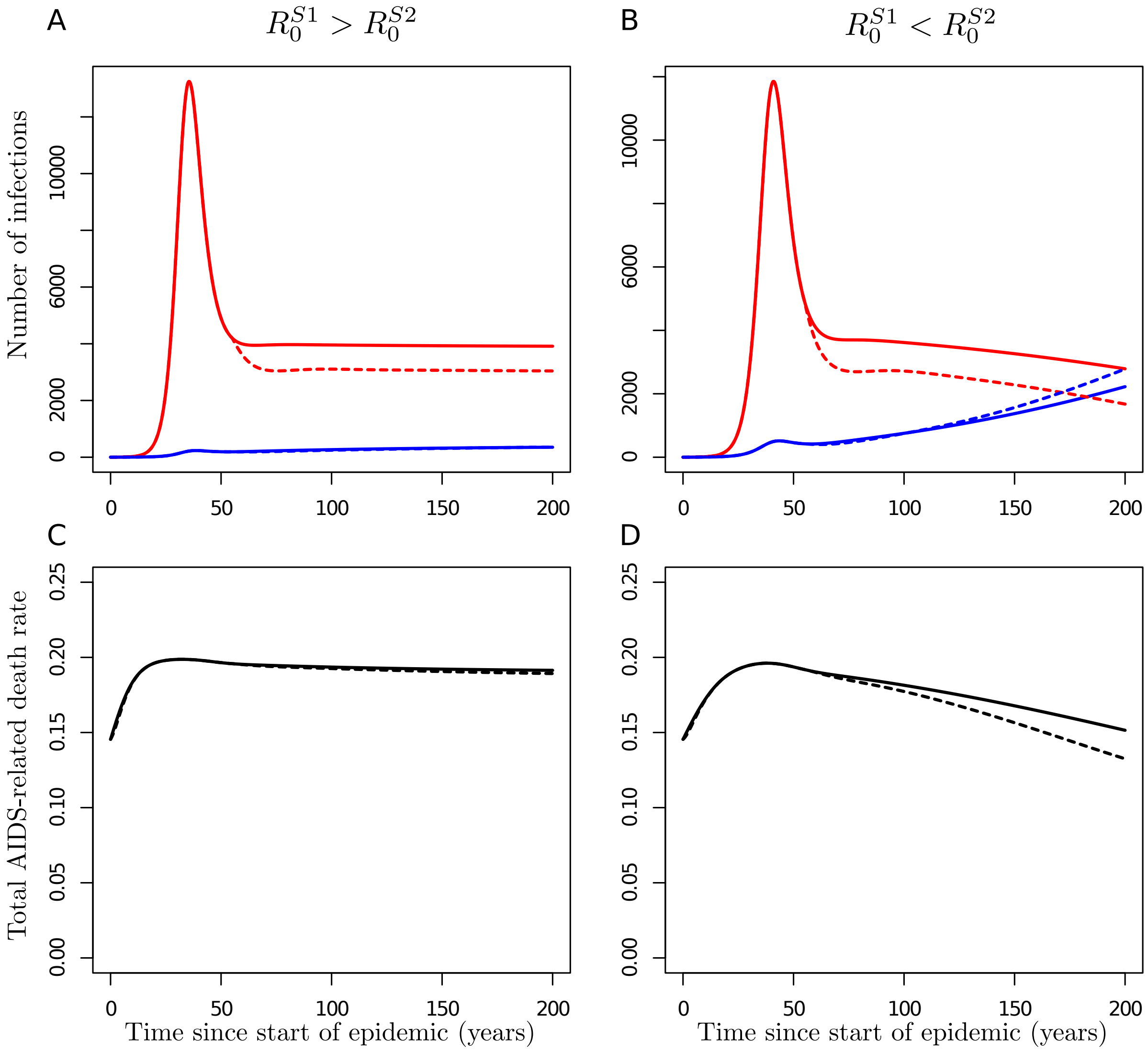

Figure S15:**Behaviour of the model without ART compared to the model where ART is introduced after the epidemic peak.** Parameters and initial conditions are as in table 1 and figure 3 (main text), including R_0 values. Solid lines show the output of the model without ART while dotted lines plot trajectories for the model when ART is introduced at 30% coverage 55 years into the epidemic. The number of strain S1 infections through time is shown in red and the number of strain S2 infections in blue. Despite ART being introduced 15 years later, patterns of virulence change in this simulation, measured as total AIDS-related deaths per untreated infected person per year, are almost identical to those in figure 3 (main text).

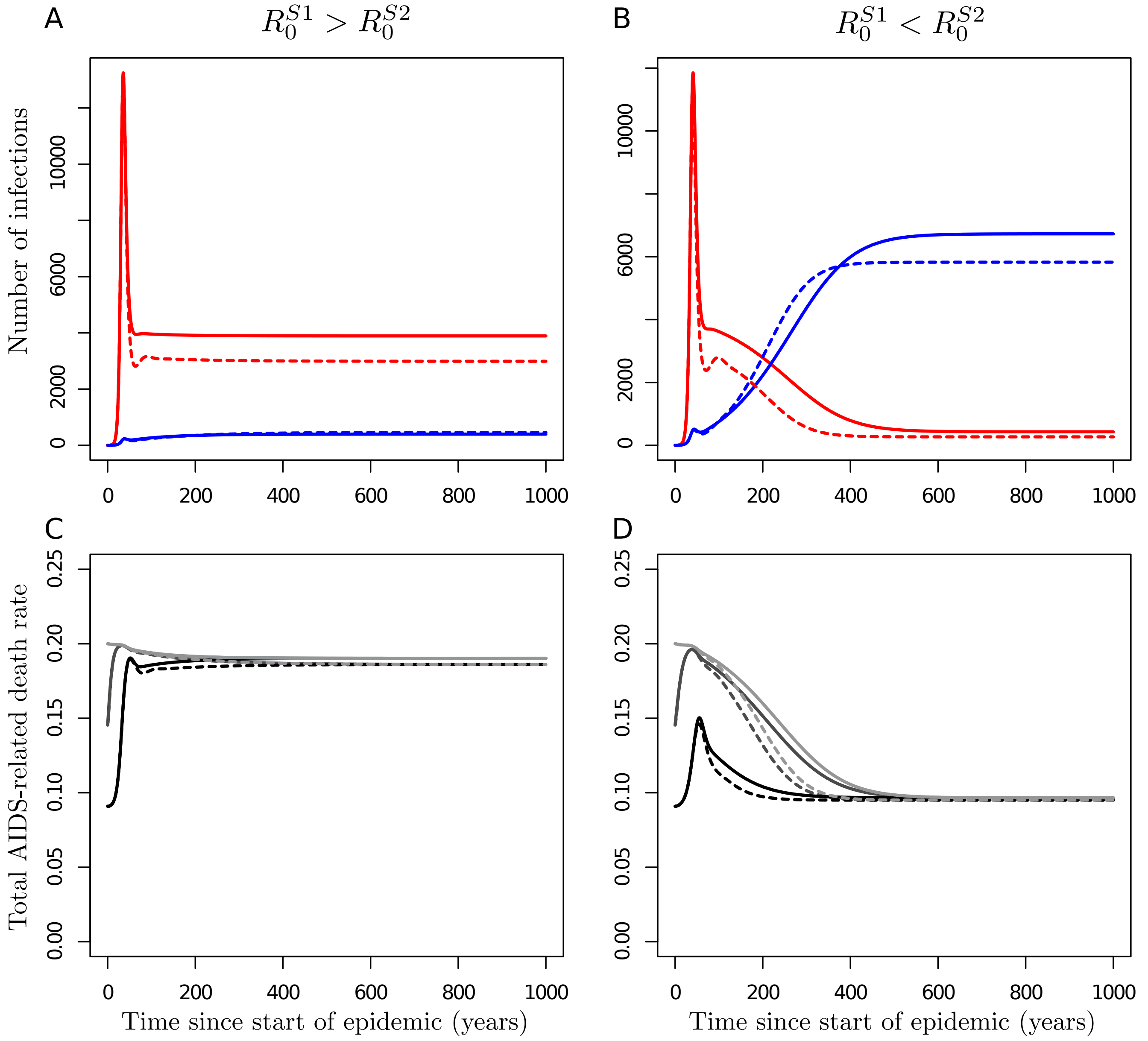

Figure S16: **Behaviour of the two strain model with and without ART and with differing initial conditions.** Parameters are the same as in figure 3 (main text) but the model is run for 1000 years in order to show the epidemic approaching equilibrium. A, B: The number of strain S1 infections through time is shown in red and the number of strain S2 infections in blue. The initial conditions are $S(0) = 40000, I^H_{S1,u}(0) = 1, I^L_{S2,u}(0) = 1$, all other populations equal to zero. C, D: The effect of three different sets of initial conditions on the average death rate of untreated individuals is shown (i) $I^H_{S1,u}(0) = 0, I^L_{S2,u}(0) = 2$ (black), (ii) $I^H_{S1,u}(0) = 1, I^L_{S2,u}(0) = 1$ (dark grey), (iii) $I^H_{S1,u}(0) = 2, I^L_{S2,u}(0) = 0$ (light grey). The initial conditions do not change the equilibrium position of the model. For (ii) and (iii) the model behaviour is close to identical after the peak of the epidemic, approximately 50 years in. For (i), when all infections are initially of the low virulence strain (S2) the model reaches equilibrium faster but the same 'second phase' patterns of decreasing virulence after the peak are still seen in both (C) and (D).

	Effect of amended model on... (figure in main text for comparison)		
Amendment (supplementary figures)	Patterns of virulence change in absence of ART (fig 3, solid lines)	Magnitude and direction of 'early' ART-effect (fig 6)	Magnitude and direction of 'late' ART-effect (fig 5)
50% of transmissions occur in acute infection (1-3)	Similar patterns seen but twice as fast (epidemic peaks earlier).	Magnitude smaller. ART more often benefits high virulence strain.	Large effects rarer. Fewer cases of decrease in virulence in particular.
Low heritability (4-6)	Clearer decrease in virulence after peak. Smaller magnitude of changes in virulence in general.	Magnitude smaller. Direction largely unchanged.	Magnitude smaller. Direction unchanged.
Core-periphery model, generalised epidemic (7)	Very similar patterns, peak slightly earlier, progressing to equilibrium slightly faster.	Slightly larger magnitude since treatment concentrated on core. Direction still negative.	Magnitude slightly larger for lower coverages. Direction unchanged.
Core-periphery model, concentrated epidemic (8)	Virulence changes less pronounced.	Larger magnitude since treatment concentrated on core. Direction still negative.	Magnitude larger for lower coverages. Direction similar.
ART initiated as soon as possible (9-10)	n/a - unchanged.	Magnitude similar in range for this range of parameters. Direction: Sometimes an increase in virulence when ART initiation is later.	Larger magnitude possible at low coverage. Direction: Always an increase in virulence.
High R_0s (11)	Similar patterns seen but twice as fast.	Magnitude slightly larger. Direction still negative.	Magnitude and direction largely unchanged (still determined by equation 8, main text).
Low R_0s (12)	Similar patterns but epidemic progresses much slower.	Magnitude and direction seem similar.	Magnitude and direction unchanged.
ART introduced 15 years before peak (14)	n/a - unchanged.	Negligible change in magnitude and direction.	n/a - unchanged.
ART introduced 15 years after peak (15)	n/a - unchanged.	Negligible change in magnitude and direction.	n/a - unchanged.
Varying initial conditions (16)	First phase (before peak) varies depending on initial conditions. Second phase decrease sometimes shorter in duration.	Magnitude can be smaller. Direction unchanged.	n/a - unchanged.

Table 1: A table of the various amendments and modifications to the original model and their effect on the main results.
